# A minimalistic model of bias, polarization and misinformation in social networks

**DOI:** 10.1038/s41598-020-62085-w

**Published:** 2020-03-26

**Authors:** Orowa Sikder, Robert E. Smith, Pierpaolo Vivo, Giacomo Livan

**Affiliations:** 1 Department of Computer Science, University College London, Gower Street, London, WC1E 6EA UK; 2 Department of Mathematics, King’s College London, Strand, London, WC2R 2LS UK; 3 Systemic Risk Centre, London School of Economics and Political Sciences, Houghton Street, London, WC2A 2AE UK

**Keywords:** Complex networks, Computational science

## Abstract

Online social networks provide users with unprecedented opportunities to engage with diverse opinions. At the same time, they enable confirmation bias on large scales by empowering individuals to self-select narratives they want to be exposed to. A precise understanding of such tradeoffs is still largely missing. We introduce a social learning model where most participants in a network update their beliefs unbiasedly based on new information, while a minority of participants reject information that is incongruent with their preexisting beliefs. This simple mechanism generates permanent opinion polarization and cascade dynamics, and accounts for the aforementioned tradeoff between confirmation bias and social connectivity through analytic results. We investigate the model’s predictions empirically using US county-level data on the impact of Internet access on the formation of beliefs about global warming. We conclude by discussing policy implications of our model, highlighting the downsides of debunking and suggesting alternative strategies to contrast misinformation.

## Introduction

We currently live in a paradoxical stage of the information age. The more we gain access to unprecedented amounts of knowledge thanks to digital technologies, the less our societies seem capable of discerning what is true from what is false, even in the presence of overwhelming evidence in support of a particular position. For example, large segments of our societies do not believe in the reality of climate change^1^ or believe in the relationship between vaccinations and autism^2^.

As recent studies indicate, over two-thirds of US adults get information from online and social media, with the proportion growing annually^3,4^. Hence, the impact such media have in shaping societal narratives cannot be understated. Online media empower their users to choose the news sources they want to be exposed to. This, in turn, makes it easier to restrict exposure only to narratives that are congruent to pre-established viewpoints^5–8^, and this positive feedback mechanism is further exacerbated by the widespread use of personalized news algorithms^9^. In other words, *confirmation bias*^10,11^ is enabled at unprecedented scales^12^.

Another major impact of digital technologies has been the increase in connectivity fostered by the growth of online social networks, which plays a double-edged role. On the one hand, it can compound the effects of confirmation bias, as users are likely to re-transmit the same information they are selectively exposed to, leading to fragmented societies that break down into online “echo chambers” where the same opinions keep being bounced around^12,13^. On the other hand, it also translates into a potentially increased heterogeneity of the information and viewpoints users are exposed to^14,15^.

Online social networks can therefore both improve and restrict the diversity of information individuals engage with, and their *net* effect is still very much debated. Empirical research is still in its infancy, with evidence for both positive and negative effects being found^15–17^. The theoretical literature is lagging somewhat further behind. While there exist a plethora of models related to information diffusion and opinion formation in social networks, a sound theoretical framework accounting for the emergence of the phenomena that are relevant to modern information consumption (rather than explicitly introducing them ad hoc), is still largely lacking.

In bounded confidence models^18–20^ agents only interact with others sharing similar opinions, and thus are characterized by a form of confirmation bias. In such models polarization is a natural outcome assuming agents are narrow enough in their choice of interaction partners^21^. However, these models tend to lack behavioural micro-foundations and a clear mechanism to link information diffusion to opinion formation, making it hard to draw conclusions about learning and accuracy amongst agents.

Social learning models^22–24^ provide a broader, empirically grounded, and analytically tractable framework to understand information aggregation^25^. Their main drawback, however, is that by design they tend to produce long run population consensus, hence fail to account for any form of opinion heterogeneity or polarization^26^. Polarization can be generated by introducing “stubborn” agents that remain fully attached to their initial opinions rather than interacting and learning from their neighbors^27,28^, a mechanism reminiscent of confirmation bias. However, the conditions under which polarization occurs are very strict, as populations converge towards consensus as soon as stubborn agents accept even a negligible fraction of influence from their neighbors^26^. A similar phenomenon is explored in the social physics literature where it is referred to as networks with “zealots”, which similarly impede consensus, such as in Ref. ^29^. A key distinction in the model we introduce in the following is that all agents are free to vary their opinions over time, resulting in cascade dynamics that separate consensus and polarization regimes.

Overall, while it is clear from the literature that some notion of “bias” in networks is a key requirement to reproduce realistic dynamics of opinion formation, it is still difficult to provide a unified framework that can account for information aggregation, polarization and learning. The purpose of the present paper is to develop a framework that naturally captures the effect of large-scale confirmation bias on social learning, and to examine how it can drastically change the way a networked, decentralized, society processes information. We are able to provide analytic results at all scales of the model. At the macroscopic scale, we determine under what conditions the model ends up in a polarised state or cascades towards a consensus. At the mesoscopic scale, we are able to provide an intuitive characterization of the trade-off between bias and connectivity in the context of such dynamics, and explain the role echo chambers play in such outcomes. At the microscopic scale, we are able to study the full distribution of each agent’s available information and subsequent accuracy, and demonstrate that small amounts of bias can have positive effects on learning by preserving information heterogeneity. Our model unveils a stylized yet rich phenomenology which, as we shall discuss in our final remarks, has substantial correspondence with the available empirical evidence.

## Results

### Social learning and confirmation bias

We consider a model of a social network described by a graph $$G=(V,E)$$ consisting of a set of agents $$V$$ (where $$| V| $$ = $$n$$), and the edges between them $$E$$. Each agent seeks to learn the unobservable ground truth about a binary statement $$X$$ such as, e.g., “global warming is/is not happening” or “gun control does/does not reduce crime”. The value $$X=+\,1$$ represents the statement’s true value, whose negation is $$X=-\,1$$.

Following standard social learning frameworks^25^, at the beginning of time ($t=0$), each agent $i$ ($i=\,1,\ldots ,n$) independently receives an initial signal ${s}_{i}=\pm \,1$, which is informative of the underlying state, i.e. $p={\rm{Prob}}({s}_{i}=+\,1| X=+\,1)=\,1-{\rm{Prob}}({s}_{i}=-\,1| X=+\,1) >$1/2. Signals can be thought of as news, stories, quotations, etc., that support or detract from the ground truth. The model evolves in discrete time steps, and at each time step $t > 0$ all agents synchronously share with their neighbors the full set of signals they have accrued up to that point. For example, the time $t=\,1$ information set of an agent $i$ with two neighbors $j$ and $\ell$ will be ${s}_{i}(t=\,1)=\{{s}_{i},{s}_{j},{s}_{\ell }\}$, their time $t=2$ set will be ${s}_{i}(t=2)=\{{s}_{i},{s}_{i},{s}_{i},{s}_{j},{s}_{j},{s}_{\ell },{s}_{\ell },{s}_{d=2}\}$, where ${s}_{d=2}$ denotes the set of all signals incoming from nodes at distance $d=2$ (i.e., $j$’s and $\ell$’s neighbors), and so on. Furthermore, we define the following:

1 $${x}_{i}(t)=\frac{{N}_{i}^{+}(t)}{{N}_{i}^{+}(t)+{N}_{i}^{-}(t)},$$

where ${N}_{i}^{+}(t)$ and ${N}_{i}^{-}(t)$ denote, respectively, the number of positive and negative signals accrued by $i$ up to time $t$. We refer to this quantity as an agent’s *signal mix*, and we straightforwardly generalize it to any set of agents $C\subseteq V$, i.e., we indicate the fraction of positive signals in their pooled information sets at time $t$ as ${x}_{C}(t)$. The list of all agents’ signal mixes at time $t$ is vectorised as $x(t)$.

Each agent forms a posterior belief of the likelihood of the ground truth given their information sets using Bayes’ rule. This is done under a bounded rationality assumption, as the agents fail to accurately model the statistical dependence between the signals they receive, substituting it with a naive updating rule that assumes all signals in their information sets to be independent ($${\rm{Prob}}(X| {s}_{i}(t))={\rm{Prob}}({s}_{i}(t)| X){\rm{Prob}}(X)$$/$${\rm{P}}{\rm{r}}{\rm{o}}{\rm{b}}({s}_{i}(t))$$, where $${\rm{Prob}}({s}_{i}(t)| X)$$ is computed as a factorization over probabilities associated to individual signals, i.e. $${\rm{Prob}}({s}_{i}(t)| X)={\prod }_{c}{\rm{Prob}}\left({s}_{i}^{(c)}(t)| X\right)$$, where $${s}_{i}^{(c)}(t)$$ denotes the *c*th component of the vector), which is a standard assumption of social learning models^25^. Under such a framework (and uniform priors), the best guess an agent can make at any time over the statement $$X$$ given their information set is precisely equal to their orientation $${y}_{i}(t)$$, where $${y}_{i}(t)=+\,1$$ for $${x}_{i}(t) > $$1/2 and $${y}_{i}(t)=-\,1$$ for $${x}_{i}(t) < $$1/2 (without loss of generality, in the following we shall choose network structures that rule out the possibility of $${x}_{i}(t)=$$1/2 taking place). The orientations of all $$n$$ agents at time $$t$$ are vectorized as $$y(t)$$; the fraction of positively oriented agents in a group of nodes $$C\subseteq V$$ is denoted as $${y}_{C}(t)$$.

The *polarization* ${z}_{C}(t)=\min ({y}_{C}(t),1\,-{y}_{C}(t))$ of the group $C$ is then defined as the fraction of agents in that group that have the minority orientation. Note that polarization equals zero when there is full consensus and all agents are either positively or negatively oriented. It is maximized when there are exactly half the group in each orientation.

It is useful to think of $x(t)$, $y(t)$ and ${z}_{V}(t)$ as respectively representing the pool of available signals, the conclusions agents draw on the basis of the available signals, and a summary measure of the heterogeneity of agents’ conclusions. In the context of news diffusion, for example, they would represent the availability of news of each type across agents, the resulting agents’ opinions on some topic, and the extent to which those opinions have converged to a consensus.

We distinguish between two kinds of agents in the model: *unbiased* agents and *biased* agents. Both agents share signals and update their posterior beliefs through Bayes’ rule, as described in the previous section. However, they differ in how they acquire incoming signals. Unbiased agents accept the set of signals provided by their neighbors without any distortion. On the other hand, biased agents exercise a model of *confirmation bias*^11,30^, and are able to distort the information sets they accrue. We denote the two sets of agents as ${\mathscr{U}}$ and ${\mathscr{B}}$, respectively.

To describe the behaviour of these biased agents we use a slight variation of the confirmation bias model introduced by Rabin and Shrag^31^. We refer to an incoming signal $s$ as *congruent* to $i$ if it is aligned with $i$’s current orientation, i.e. if $s={y}_{i}(t)$, and *incongruent* if $s=-\,{y}_{i}(t)$. When biased agents are presented with incongruent signals, they reject them with a fixed probability $q$ and replace them with a congruent signal, which they add to their information set and propagate to their neighbors. We refer to $q$ as the *confirmation bias* parameter. Denote the set of positively (negatively) oriented biased agents at time $t$ as ${{\mathscr{B}}}^{+}(t)$ (${{\mathscr{B}}}^{-}(t)$), and the corresponding fraction as ${y}_{{\mathscr{B}}}^{+}(t)=| {{\mathscr{B}}}^{+}(t)|$/$| {\mathscr{B}}|$. Note that this is an important departure from “stubborn agent” models, as such biased agents do have a non-zero influence from their neighbors, and they can change their beliefs over time as they aggregate information.

**Figure 1:**
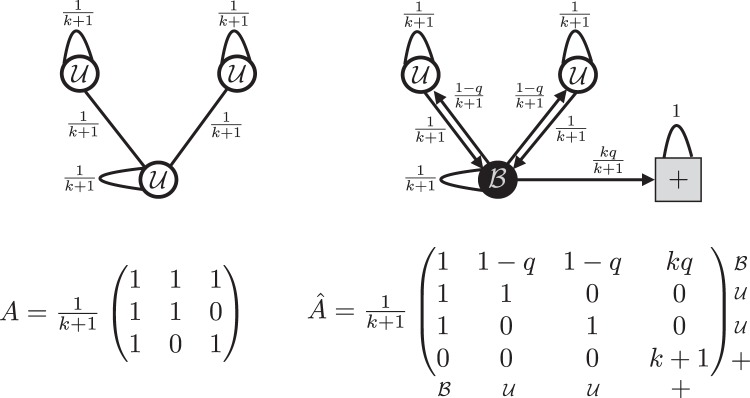
Sketch of an unbiased (left) and biased (right) network with three nodes. Below each sketch is the update matrix of the corresponding (deterministic) DeGroot model. The network on the right shows that the stochastic “signal distortion” behaviour can be approximated with a biased agent (${\mathscr{B}}$) reducing the weight it places on each of its neighbors to $(1-q)$/$(k+\,1)$, and placing the remaining weight $kq/(k+\,1)$ on an external positively oriented “ghost” node. When a biased agent changes orientation, it switches such an edge to an external negatively oriented node instead.

An intuitive interpretation of what this mechanism is intended to model is as follows: biased agents are empowered to reject incoming signals they disagree with, and instead refer to preferred sources of information to find signals that are congruent with their existing viewpoint (see Fig. 1). This mechanism models both *active* behaviour, where agents deliberately choose to ignore or contort information that contradicts their beliefs (mirroring the “backfire effect” evidenced both in psychological experiments^32,33^ and in online social network behaviour^34^), and *passive* behaviour, where personalized news algorithms filter out incongruent information and select other information which coheres with the agents’ beliefs^15^.

In the following, we shall denote the fraction of biased agents in a network as $f$. We shall refer to networks where $f=0$ as *unbiased* networks, and to networks where $f > 0$ as *biased* networks.

For the bulk of the analytic results in the paper, we assume that the social network $G$ is an undirected $k$-regular network. The motivation for this is two-fold. Firstly, empirical research^35^ suggests that for online social networks such as Facebook (where social connections are symmetric), heterogenous network features such as hubs do not play a disproportionately significant role in the diffusion of information. Intuitively, while social networks themselves might be highly heterogenous, the network of information transmission is a lot more restricted, as individuals tend to discuss topics with a small group such as immediate friends and family. Secondly, utilizing a simple $k$-regular network allows for considerable analytical tractability. However, one can show that our main results can be easily extended to hold under a variety of network topologies characterized by degree heterogeneity.

The assumption of regular network structure (coupled with the aforementioned synchronous belief update dynamics) allows the information sets of all agents to grow at the same rate, and as a result the evolution of the signal mix $x(t)$ can be mapped to a DeGroot averaging process for unbiased networks^36^: $x(t)=A\,x(t-\,1)$, where $A$ is an $n\times n$ matrix with entries ${a}_{ij}=\,1$/$(k+\,1)$ for each pair $(i,j)$ of connected nodes.

For biased networks, one can demonstrate (see Section S1 of the Supplementary Materials) that the above confirmation bias mechanics can be reproduced by introducing a positive and negative “ghost” node which maintain respective signal mixes of $1$ and $0$. Biased agents sample each signal from their orientation-aligned ghost node with probability $q$, and from their neighborhood with probability $(1-q)$.

Furthermore, while the process is stochastic, we also show that it converges to a deterministic process with a simple update matrix described as follows. In Section S3 of the Supplementary Materials we discuss in detail the correspondence and convergence between the stochastic and deterministic processes. Biased agents down-weight their connections to neighbors by a factor $(1-q)$ and place the remaining fraction $kq$/$(k+\,1)$ of their outgoing weight on the corresponding ghost node. With these positions the updating process now simply reads $\widehat{x}(t)={{\hat{\rm A}}}(t)\widehat{x}(t-\,1)$, where ${{\hat{\rm A}}}(t)$ is an $(n+2)\times (n+2)$ asymmetric matrix whose entries in its $n\times n$ upper-left block are as those in $A$, except ${{ {\hat{a}} }}_{ij}=(1-q)$/$(k+\,1)$ when $i\in {\mathscr{B}}={{\mathscr{B}}}^{+}\cup {{\mathscr{B}}}^{-}$. The positive (negative) ghost node corresponds to node $n+\,1$ ($n+2$) of the augmented matrix ${{\hat{\rm A}}}(t)$, and we shall label it as $+$ ($-$) for convenience, i.e., we shall have ${{ {\hat{a}} }}_{i+}=kq$/$(k+\,1)$ (${{ {\hat{a}} }}_{i-}=kq$/$(k+\,1)$) for $i\in {{\mathscr{B}}}^{+}$ ($i\in {{\mathscr{B}}}^{-}$) and ${{ {\hat{a}} }}_{++}={{ {\hat{a}} }}_{--}=\,1$. Similarly, $\widehat{x}(t)$ denotes an augmented signal mix vector where ${\widehat{x}}_{+}(t)={\widehat{x}}_{n+1}(t)=\,1$, and ${\widehat{x}}_{-}(t)={\widehat{x}}_{n+2}(t)=0$.

The time dependence of the matrix ${{\hat{\rm A}}}(t)$ is due to the fact that whenever a biased agent switches orientation its links to the ghost nodes change. This happens whenever the agent’s signal mix ${x}_{i}(t)$ (see Eq. (1)) goes from below to above 1/2 or vice versa, due to an overwhelming amount of incongruent incoming signals from its neighbors. For example, when switching from being positively to negatively oriented, a biased agent $i$ will change its links as follows: ${{ {\hat{a}} }}_{i+}=kq$/$(k+\,1)\to {{ {\hat{a}} }}_{i+}=0$, and ${{ {\hat{a}} }}_{i-}=0\to {{ {\hat{a}} }}_{i-}=kq$/$(k+\,1)$. In the following, all quantities pertaining to biased networks will be denoted with aˆ symbol.

We provide a sketch of the above mapping in Fig. 1 (for the sake of simplicity we present an arbitrary small network instead of a strictly $k$-regular one). There is an appealing intuition to this interpretation: biased agents have a “preferred” information source they sample from in lieu of incongruent information provided from their peers. If their beliefs change, their preferred information source can change.

Our main focus will be on the long-run properties of the dynamics introduced above. In this respect, it is important to establish whether the agents actually reach an equilibrium over their signal mixes and orientations, or whether they continue to oscillate. It is straightforward to demonstrate that unbiased networks always converge to a limiting steady state for their signal mixes and orientations, which follows directly from the correspondence between such networks and DeGroot models^23^. The convergence of biased networks is much less trivial to prove due to the non-linear dynamics introduced by the confirmation bias mechanics. It can be shown however that convergence holds under fairly general conditions, and in Section S2 of the Supplementary Materials we demonstrate such convergence under a number of network topologies.

Building on this, we are able to establish the following results on $k$-regular networks of any size (in the following, and throughout the rest of the paper, we shall denote the steady state value of a variable $v$ as ${v}^{\ast }$).

For biased $k$-regular networks, denote ${t}^{\ast }$ as the time after which biased agents cease switching their orientation. Define ${{\hat{Y}}}_{{\mathscr{B}}}^{\ast }$ as the steady state fraction of positively oriented biased agents. Then the following holds (see Section S3 of the Supplementary Materials).

1. The signal mix vector $\widehat{x}(t)$ converges to some ${\widehat{x}}^{\ast }={{{\hat{\rm A}}}}^{\ast }\widehat{x}(0)$ for both biased and unbiased networks, where ${{{\hat{\rm A}}}}^{\ast }$ is a steady-state matrix of influence weights which can be computed explicitly (see Section S3 of the Supplementary Materials).
2. Unbiased networks achieve consensus, and converge to influence weights of ${a}_{ij}^{\ast }=\,1$/$n$ for all pairs $(i,j)$. This ensures that, for all $i\in V$, ${x}_{i}^{\ast }={x}_{V}^{\ast }=\bar{x}(0)$, where $\bar{x}(0)={n}^{-1}{\sum }_{i=\,1}^{n}{s}_{i}$ is the intial average signal mix.
3. Biased networks where ${{\hat{Y}}}_{{\mathscr{B}}}^{\ast }=0,1$ achieve consensus, and converge to influence weights ${{ {\hat{a}} }}_{ij}^{\ast }=0$ for all pairs $(i,j)\in V$, ${{ {\hat{a}} }}_{i+}^{\ast }={{\hat{Y}}}_{{\mathscr{B}}}^{\ast }$ and ${{ {\hat{a}} }}_{i-}^{\ast }=\,1-{{\hat{Y}}}_{{\mathscr{B}}}^{\ast }$ for all $i\in V$.
4. Biased networks where $0 < {{\hat{Y}}}_{{\mathscr{B}}}^{\ast } < 1$ do not achieve consensus, and converge to influence weights ${{ {\hat{a}} }}_{ij}^{\ast }=0$ for all $(i,j)\in V$, and ${{ {\hat{a}} }}_{i+}^{\ast }+{{ {\hat{a}} }}_{i-}^{\ast }=\,1$ for all $i\in V$.

From the above, we can conclude that while unbiased networks efficiently aggregate the information available to them at $t=0$, the outcome of the information aggregation process in biased networks ends up being *entirely* determined by the long-run orientations of biased agents. We shall devote the following sections to examine the consequences of the model in greater detail through mean field approximations coupled with numerical verifications on finite networks.

### Cascades and consensus

We begin by studying the signal mix of unbiased agents in biased networks (${\widehat{x}}_{{\mathscr{U}}}^{\ast }$) to provide a like for like comparison with the fully unbiased networks. In the context of the diffusion of news, the global signal mix can be thought of as a model of the long term balance of news of different types that survive following the diffusion dynamics.

For unbiased networks, it is demonstrated in^37^ that ${\widehat{x}}_{{\mathscr{U}}}^{\ast }=\bar{x}(0)$. That is, the steady state signal mix in unbiased networks precisely reflects the original, unbiased informative signals injected into the network. Determining the steady state signal mix of biased networks entails considering the interactions between three subpopulations - the unbiased agents ${\mathscr{U}}$, positively biased agents ${{\mathscr{B}}}^{+}$, and negatively biased agents ${{\mathscr{B}}}^{-}$. One can show (see Section S4 of the Supplementary Materials) that this can be approximated as:

2 $$\left(\begin{array}{c}{\widehat{x}}_{{\mathscr{U}}}^{\ast }\\ {\widehat{x}}_{{{\mathscr{B}}}^{+}}^{\ast }\\ {\widehat{x}}_{{{\mathscr{B}}}^{-}}^{\ast }\end{array}\right)=\left(\begin{array}{c}{{\hat{Y}}}_{{\mathscr{B}}}({t}^{\ast })\\ (1-q){{\hat{Y}}}_{{\mathscr{B}}}({t}^{\ast })+q\\ (1-q){{\hat{Y}}}_{{\mathscr{B}}}({t}^{\ast })\end{array}\right).$$

Let us now consider the situation under which ${t}^{\ast }=0$, i.e. where the initial orientation of each biased agent does not change, and is therefore equal to the initial signal it receives. Intuitively, this will occur for large $q$ which allows for biased agents to reject the majority of incongruent signals they receive (shortly we demonstrate in fact this generally occurs for $q\, > \,1$/$2$). Given Eq. (2), we can therefore calculate the steady state signal mix of any subset of agents based on our knowledge of the distribution of the initial signals.

In this scenario, the average signal mix ${\widehat{x}}_{{\mathscr{U}}}^{\ast }$ of unbiased agents is determined by the initial proportion of positively oriented biased agents ${y}_{{{\mathscr{B}}}^{+}}(0)$, which is the mean of $fn$ i.i.d. Bernoulli variables with probability $p$ (which, we recall, denotes the probability of an initially assigned signal being informative). One can compare this to unbiased networks ($f=0$), where the long run average signal mix is ${x}_{V}^{\ast }=\bar{x}(0)$, and is hence the mean of $n$ i.i.d. Bernoulli variables with probability $p$. Applying the central limit theorem we see that injecting a fraction $f$ of biased agents therefore amplifies the variance of the long run global signal mix by a factor of ${f}^{-1}$ with respect to the unbiased case:

3 $${x}_{V}^{\ast } \sim {\mathscr{N}}\left(p,\frac{p(1-p)}{n}\right)\to {\widehat{x}}_{{\mathscr{U}}}^{\ast } \sim {\mathscr{N}}\left(p,\frac{p(1-p)}{fn}\right).$$

This means that the “wisdom of unbiased crowds” is effectively undone by small biased populations, and the unbiased network’s variability is recovered for $f\to 1$, and not for $f\to {0}^{+}$, as one might intuitively expect.

Consider now the general case where biased agents can, in principle, switch orientation a few times before settling on their steady state orientation. Using mean-field methods one can determine the general conditions under which a cascade in these orientation changes can be expected (see Section S4 of the Supplementary Materials) but here we only provide some intuition. As $q$ is lower, it is easier for an initial majority camp of biased agents to convert the minority camp of biased agents. As the conversion of the minority camp begins, this triggers a domino effect as newly converted biased agents add to the critical mass of the majority camp and are able to overwhelm the minority orientation.

This mechanism allows us to derive analytic curves in the parameter space to approximate the steady state outcome of the unbiased agent population’s average signal mix based on the orientations of the biased agents at time $0$:

4 $${\widehat{x}}_{{\mathscr{U}}}^{\ast }=\left\{\begin{array}{ll}{{\hat{Y}}}_{{\mathscr{B}}}(0) & \,{\rm{for}}\,\,\frac{1-2q}{2(1-q)}\le {{\hat{Y}}}_{{\mathscr{B}}}(0)\le \frac{1}{2(1-q)}\\ 1 & \,{\rm{for}}\,\,{{\hat{Y}}}_{{\mathscr{B}}}(0) > \frac{1-2q}{2(1-q)}\\ 0 & \,{\rm{for}}\,\,{{\hat{Y}}}_{{\mathscr{B}}}(0) < \frac{1}{2(1-q)}.\end{array}\right.$$

**Figure 2:**
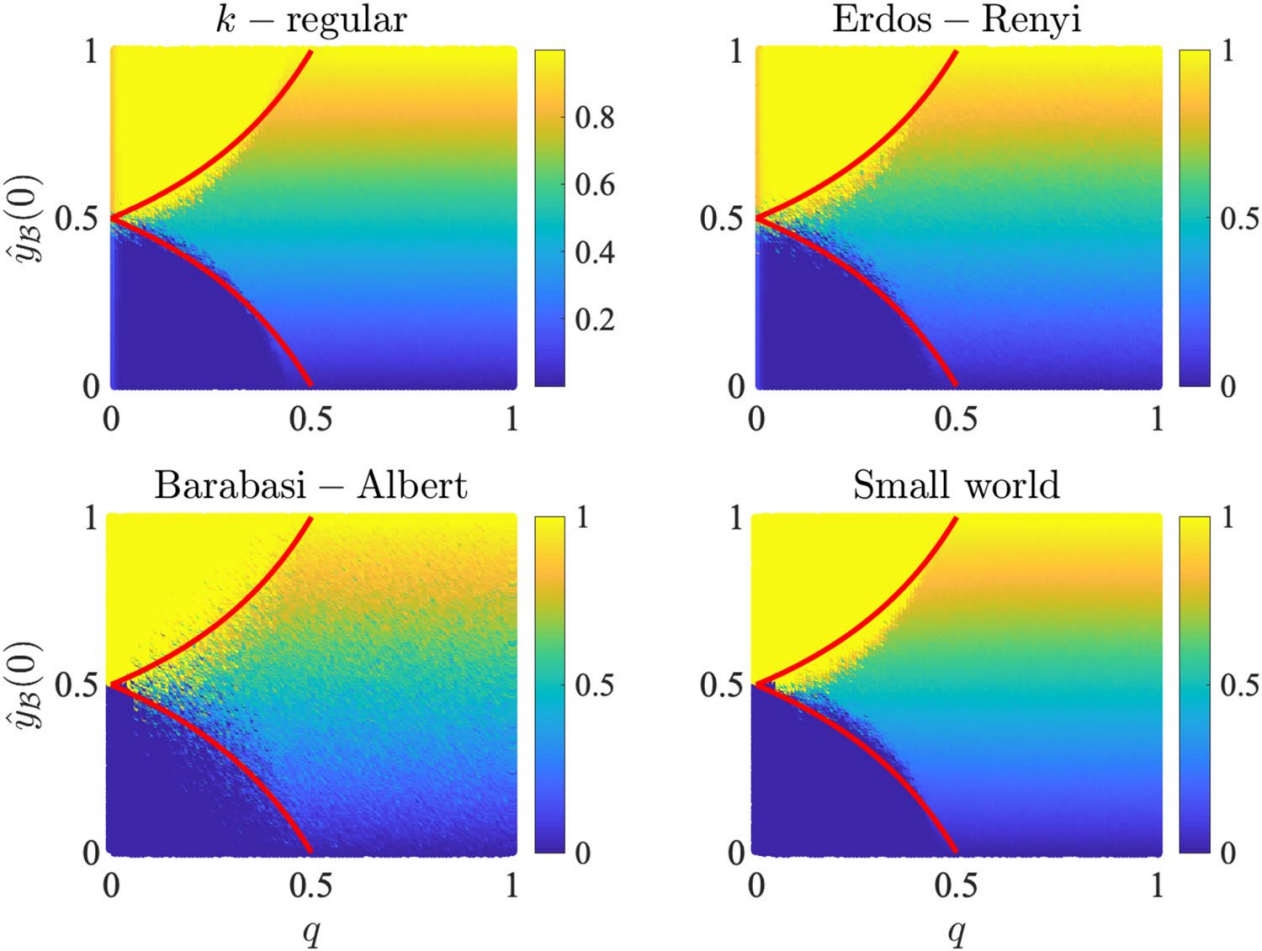
Average steady state signal mix of unbiased agents (${\widehat{x}}_{{\mathscr{U}}}^{\ast }$) as a function of the time $t=0$ fraction of positively oriented biased agents (${{\hat{Y}}}_{{\mathscr{B}}}(0)$) and confirmation bias $q$. The color gradient denotes the average long run signal mix for unbiased agents from $0$ to $1$. The top-left and bottom-left regions are characterized, respectively, by a global signal mix of 1 and 0 respectively, and are separated by a discontinuous transition from a region characterized by a steady state that maintains a mixed set of signals. That is, in the top-left region almost all negative signals have been removed from the network, leaving almost entirely positive signals in circulation (and vice versa for the bottom-left region). In the remaining region, signal mixes of both types survive in the long run, and the balance between positive and negative signals reflects the fraction of positively oriented biased agents. The lower $q$ falls, the easier it is to tip the network into a total assimilation of a single signal type. Results are shown for simulations on $k$-regular (top left), Erdős-Rényi (top right), Barabasi-Albert (bottom left) and Small-world (bottom right) networks. Analytic predictions (given by Eq. (4)) are denoted by solid red lines. The parameters used in the simulations were $n=\,1{0}^{3}$, $p=0.51$, $k=6$ (which corresponds to an average degree in all cases), $f=0.4$.

The above result is sketched in Fig. 2, and we have verified that it matches numerical simulations even for heterogenous networks. For 1/2 $< q\le 1$ biased agents can convert at least half of the incongruent signals they receive to their preferred type, meaning that biased agents of either orientation cannot be eradicated from the network, which preserves signals of both types in the steady state. For sufficiently small values of $q$, on the other hand, small variations in the initial biased population translate to completely opposite consensus, and only by increasing the confirmation bias $q$, paradoxically, the model tends back to a balance of signals that resembles the initially available information.

Putting the above results together, we note that biased networks with small $f$ and $q$ are, surprisingly, the most unstable. Indeed, such networks sit on a knife-edge between two extremes where one signal type flourishes and the other is totally censored. In this context, the model indicates that confirmation bias helps preserve a degree of information heterogeneity, which, in turn, ensures that alternative viewpoints and information are not eradicated. In subsequent sections we consider a normative interpretation of this effect in the context of accuracy and learning.

### Polarization, echo chambers and the bias-connectivity trade-off

So far we have derived the statistical properties of the average steady state signal mix across *all* unbiased agents. We now aim to establish how these signals are distributed *across* individual agents. Throughout the following, assume the global steady state signal mix ${\widehat{x}}_{{\mathscr{U}}}^{\ast }$ has been determined.

**Figure 3:**
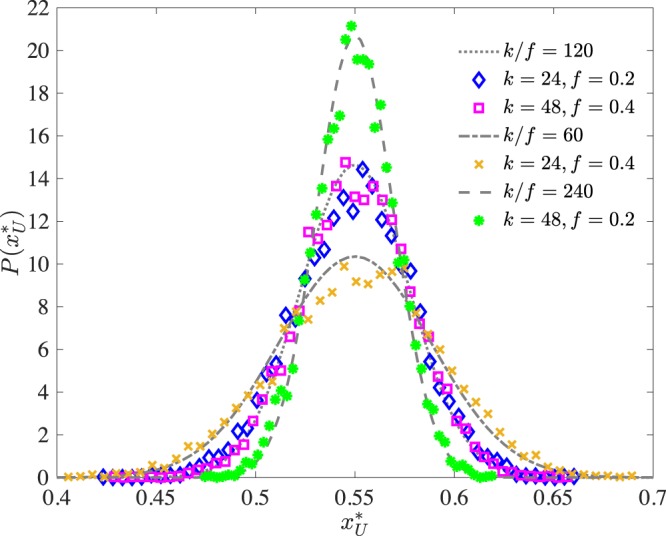
Distribution of individual unbiased agents’ steady state signal mixes (points) vs analytic predictions (dashed lines). As discussed in the main text, the model predicts such distribution to be a Gaussian (for both large $n$ and $k$) with mean equal to ${\widehat{x}}_{{\mathscr{U}}}^{\ast }$ and variance given by Eq. (5). ${\widehat{x}}_{{\mathscr{U}}}^{\ast }$ is kept fixed to demonstrate the effect of varying $f$ and $k$. As shown in the case for $k/f=120$, $f$ and $k$ trade off, and scaling both by the same constant results in the same distribution. The parameters used in the simulations were $n=\,1{0}^{4}$, ${\widehat{x}}_{{\mathscr{U}}}^{\ast }=0.55$, $q=0.6$.

In the limit of large $n$ and $k$, ${x}_{i}^{\ast }$ for $i\in {\mathscr{U}}$ is normally distributed with mean ${\widehat{x}}_{{\mathscr{U}}}^{\ast }$ and variance ${\sigma }^{2}({\widehat{x}}_{{\mathscr{U}}}^{\ast })$ that can be approximated as follows (see Section S4 of the Supplementary Materials):

5 $${\sigma }^{2}({\widehat{x}}_{{\mathscr{U}}}^{\ast })\approx \frac{f{q}^{2}}{k}({\widehat{x}}_{{\mathscr{U}}}^{\ast }(1-{\widehat{x}}_{{\mathscr{U}}}^{\ast })),$$

and this result is quite accurate even when compared with simulations for small $n$ and $k$, as demonstrated in Fig. 3.

**Figure 4:**
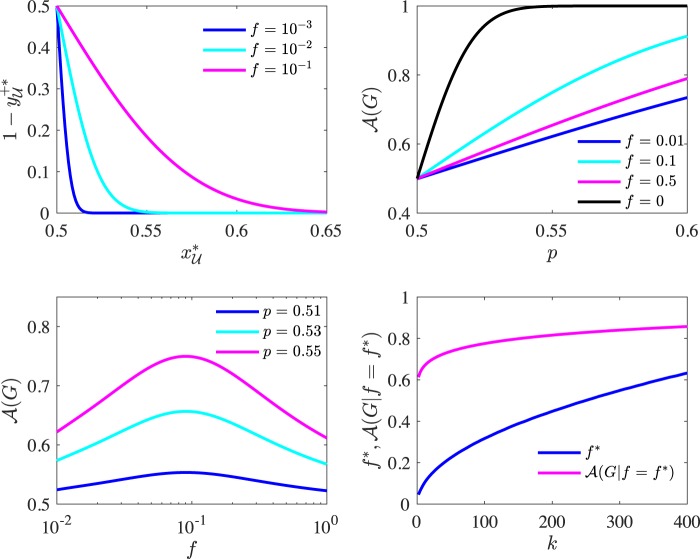
(Top left panel) Polarization ${ {\hat{z}} }_{{\mathscr{U}}}^{\ast }=\,1-{{\hat{Y}}}_{{\mathscr{U}}}^{+\ast }$ of the unbiased agent population as a function of the average signal mix ${\widehat{x}}_{{\mathscr{U}}}^{\ast }$ in the steady state calculated as ${\rm{erfc}}\left.\left({\widehat{x}}_{{\mathscr{U}}}^{\ast }-\,1/2\right)/\left(\sqrt{2}\sigma \left({\widehat{x}}_{{\mathscr{U}}}^{\ast }\right)\right)\right)$/2, with $\sigma ({\widehat{x}}_{{\mathscr{U}}}^{\ast })$ given by Eq. (5) . (Top right panel) Expected accuracy (Eq. (6)) as a function of the initial signals’ informativeness $p$. (Bottom left panel) Expected accuracy (Eq. (6)) as a function of the fraction $f$ of biased agents. (Bottom right panel) Behaviour of the accuracy-maximizing value ${f}^{\ast }$ and of the corresponding accuracy ${\mathscr{A}}(G| f={f}^{\ast })$ as functions of $k$ . In the first three panels the model’s parameters are $n=\,1{0}^{3}$, $q=\,1$, $k=8$, while the parameters in the last panel are $n=\,1{0}^{3}$, $q=\,1$, $p=0.53$. In all cases we assume $X=+\,1$ without loss of generality.

This result further shows that the presence of biased agents is effectively responsible for the polarization of unbiased agents in the steady state. Indeed, both a larger biased population and higher confirmation bias - i.e. higher $f$ or $q$, respectively - result in an increased variance and steady state polarization ${ {\hat{z}} }_{{\mathscr{U}}}^{\ast }$, since a larger variance ${\sigma }^{2}({x}_{{\mathscr{U}}}^{\ast })$ implies larger numbers of agents displaying the minority orientation. This is illustrated in the top left panel of Fig. 4.

**Figure 5:**
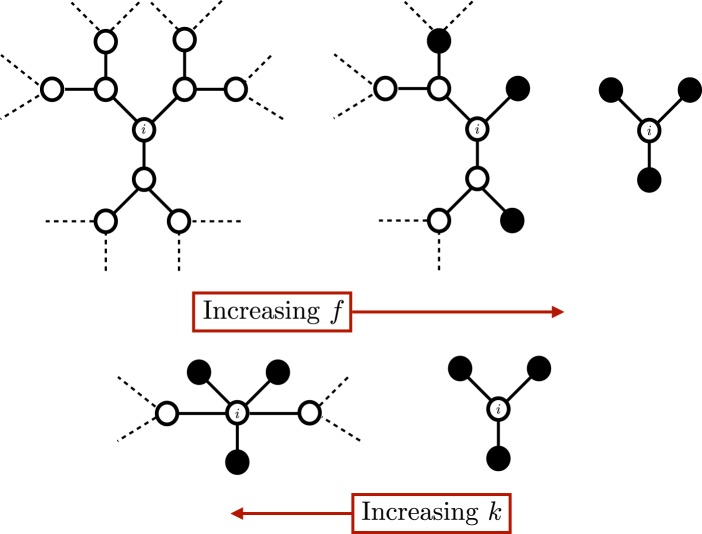
For illustration of the bias-connectivity trade-off, consider a Cayley $3$-tree structure and let $q=\,1$. When $f=0$, each unbiased node has 3 independent sources of novel information, one from each branch associated with a neighbor. As $f$ increases, unbiased neighbors are steadily replaced with biased agents that act as gatekeepers and restrict the flow of novel information from their branches, resulting in an equivalence with fewer branches overall. Increasing social connectivity $k$ bypasses biased neighbors and enables the discovery of novel information. This can also be interpreted as agents being encased in “echo chambers” as bias grows, and circumventing these chambers as connectivity increases.

On the other hand, a larger degree $k$ contrasts this effect by creating more paths to transport unbiased information. It is worth pointing out that the variance in Eq. (5) does not decay with $n$, showing that steady state polarization persists even in the large $n$ limit. We refer to this as the bias-connectivity trade-off, and the intuition behind this result is illustrated in Fig. 5.

Further intuition for this result can be found at the mesoscopic level of agent clusters, where we see the emergence of natural “echo chambers” in the model. We define an echo chamber $C$ as a subset of unbiased agents such that: $C=\{i\in {\mathscr{U}}:{\partial }_{i}\in {\mathscr{B}}\cup C\,\wedge {\partial }_{i}\cap C\ne {\rm{\varnothing }}\}$, where ${\partial }_{i}$ denotes the neighborhood of agent $i$. In other words, an echo chamber is a set of connected unbiased agents such that all nodes are either connected to other nodes in the echo chamber or to biased agents. Therefore, biased agents form the echo chamber’s boundary, which we refer to as ${\partial }_{C}$. Echo chambers in our model represent groups of unbiased agents that are completely surrounded by biased agents who effectively modulate the information that can flow in and out of these groups.

Echo chambers allow us to examine the qualitative effect of confirmation bias ($f,q$) and connectivity $k$. Let us label the fraction of unbiased agents enclosed in an echo chamber as ${\eta }_{C}$. Leveraging some simple results from percolation theory^38^ we can show that ${\eta }_{C}$ increases with $f$ and decreases with $k$, as the creation of more pathways that bypass biased agents effectively breaks up echo chambers. Furthermore, the equilibrium signal mix of unbiased agents inside echo chambers is well approximated by a weighted average between the signal mix of the biased agents surrounding them (${x}_{{\partial }_{C}}^{\ast }$) and the signal mix ${x}_{{\mathscr{U}}}^{\ast }$ of the whole population: ${x}_{C}^{\ast }=q\,{x}_{\partial C}^{\ast }+(1-q){x}_{{\mathscr{U}}}^{\ast }$. The confirmation bias parameter $q$ therefore determines the “permeability” of echo chambers to the information flow from the broader network. Hence, unbiased agents enclosed in echo chambers are likely to be exceedingly affected by the views of the small set of biased agents surrounding them, and, as such, to hold information sets that are unrepresentative of the information available to the broader network. In doing so, we can envision these echo chambers as effective “building blocks” of the overall polarization observed in the network.

### Accuracy, efficiency and learning

Up until now, we have not attempted to make any normative interpretations of the ground truth $X=+\,1$. In the following, we shall refer to unbiased agents whose steady state orientation is positive (negative) as accurate (inaccurate) agents, and we shall define the overall accuracy ${\mathscr{A}}(G)$ of a network $G$ as the *expected* fraction of accurate agents in the steady state. This allows us to investigate how biased and unbiased networks respond to changes in the reliability of the available information, which ultimately depends on the prevalence of positive or negative signals (modulated by the parameter $p={\rm{Prob}}(s=+\,1| X=+\,1)$), which, loosely speaking, can be interpreted as “real” and “fake” news.

The accuracy of unbiased networks obtains a neat closed form that can be approximated as ${\mathscr{A}}(G| f=0)\approx {\rm{erfc}}((1-2p)\sqrt{n/2})$/2 (see^37^). For $f > 0$, we compute the expected accuracy as the expected fraction of accurate agents with respect to a certain global signal mix. This reads:

6 $${\mathscr{A}}(G| f > 0)=\frac{1}{2}{\int }_{0}^{1}{\rm{d}}{x}_{{\mathscr{U}}}^{\ast }\,P({x}_{{\mathscr{U}}}^{\ast })\,{\rm{erfc}}\left(\frac{1/2-{x}_{{\mathscr{U}}}^{\ast }}{\sqrt{2}{\sigma }_{{x}_{{\mathscr{U}}}^{\ast }}}\right)\,,$$

where $P({x}_{{\mathscr{U}}}^{\ast })$ is the distribution of the average signal mix across unbiased agents (see Eq. (3)) (we take the simplifying case of $q\, > \,1/2$, but this can easily be extended to the case for $q\,\le \,1/2$ using Eq. (4)), and where we have used the previously mentioned Gaussian approximation for the distribution of individual signal mixes (whose variance ${\sigma }_{{x}_{{\mathscr{U}}}^{\ast }}^{2}$ is given by Eq. (5)).

The top right panel in Fig. 4 contrasts biased and unbiased networks, and shows how the former remain very inefficient in aggregating information compared to the latter, even as the reliability of the signals ($p$) improve. However, accuracy in biased networks is non-monotonic with respect to $f$. As shown in the bottom left panel in Fig. 4, accuracy reaches a maximum in correspondence of an optimal value ${f}^{\ast }$ (see Section S5 of the Supplementary Materials for a comparison with numerical simulations). Intuitively, this is because for small values of $f$, as already discussed, the model can converge to the very inaccurate views of a small set of biased agents. As $f$ grows, the views of the two biased camps tend to cancel each other out, and the signal set will match more closely the balance of the original distribution of signals (Eq. (3)). However, in doing so large values of $f$ lead to increased polarization (Eq. (5)), where accurate and inaccurate agents coexist. The trade-off between balance and polarization is optimised at ${f}^{\ast }$.

It is also interesting to note that, as shown in the bottom right panel of Fig. 4, the optimal fraction of biased agents ${f}^{\ast }$ and the corresponding maximum accuracy ${\mathscr{A}}(G| f={f}^{\ast })$ both increase monotonically with the degree. This indicates that as networks are better connected, they can absorb a greater degree of confirmation bias without affecting accuracy.

### Internet access, confirmation bias, and social learning

We now seek to test some of the model’s predictions against real world data. Clearly, a full validation of the model will require an experimental setup, but a simple test case on existing data can demonstrate the utility of the framework in disambiguating the competing effects of bias and connectivity. We employ the model to investigate the effect of online media in the process of opinion formation using survey data. Empirical literature on this phenomenon has been mixed, with different analyses reaching completely opposite conclusions, e.g., showing that Internet access increases^39^, decreases^40^ and has no effect^41^ on opinion polarization. In Section S6 of the Supplementary Materials we briefly review how our model can help better understand some of the inconsistencies between these results.

Our position is that the effect of Internet access can be split into the effect it has on social connectivity and social discussion ($k$) and the residual effect it has on enabling active and passive confirmation bias behaviours ($f$). As per Eq. (5), assuming the majority of the population accurately learns the ground truth (${x}_{{\mathscr{U}}}^{\ast } >$1/2), increases in social discussion should improve consensus around the truth and reduce the fraction of inaccurate agents. However, when controlling for the improvement in social connectivity, we should expect an increase in Internet access to have the opposite effect.

We utilise data from the Yale Programme on Climate Change Communication^42^, which provides state and county level survey data on opinions on global warming, as well as information about the propensity to discuss climate change with friends and family, which proxies connectivity $k$. We combine this with FCC reports on county level broadband internet penetration, which proxies for $f$ after controlling for the considerable effect this has on social connectivity. We also account for a range of covariates (income, age, education, etc) and make use of an instrumental variable approach to account for simultaneous causality. We then attempt to predict the fraction of each county’s population that correctly learns that “global warming is happening” (see Section S6 of the Supplementary Materials for details on assumptions and results).

As predicted by our model, we find the accurate fraction of the population to have statistically significant positive relationships with $k$, and a negative relationship with $f$. We find our model accounts for $65 \%$ of the variance in the data. This indicates that, after controlling for the improvements on social connectivity, Internet access does indeed increase polarization and reduces a population’s ability to accurately learn. While simple, this analysis illustrates the value of our model: by explicitly accounting for the separate effects of large-scale online communication (confirmation bias and connectivity), it can shed light on the mixed empirical results currently available in the literature. In Section S6 of the Supplementary Materials we explore this further by reviewing some of these empirical results and showing how our model provides useful further interpretations of available findings.

It should be emphasized that this result is merely an initial exploration of how our model can provide some testable predictions to empirical data, as opposed to a detailed effort to understand the effect of Internet access on global warming beliefs. Having said that, the initial results are encouraging, and we hope the clarity of the analytic results of our model pave the way for testing variations of the idea of biased information aggregation in a range of outcomes and settings.

## Discussion

We introduced a model of social learning in networked societies where only a fraction of the agents update beliefs unbiasedly based on the arrival of new information. The model only provides a stylized representation of the real-world complexity underpinning the propagation of information and the ensuing opinion formation process. Its value stands in the transparency of the assumptions made, and in the fact that it allows us to “unpack” blanket terms such as, e.g., social media and Internet penetration, by assigning specific parameters to their different facets, such as connectivity ($k$) and the level of confirmation bias it enables in a society ($f,q$). This, in turn, yields quantitative *testable* predictions that contribute to shed light on the mixed results that the empirical literature has so far collected on the effects online media have in shaping societal debates.

Our model indicates the possibility that the “narratives” (information sets) biased societies generate can be *entirely* determined by the composition of their sub-populations of biased reasoners. This is reminiscent of the over-representation in public discourse of issues that are often supported by small but dedicated minorities, such as GMO opposition^43^, and of the domination of political news sharing on Facebook by heavily partisan users^15^; it also resonates with recent experimental results showing that committed minorities can overturn established social conventions^44^. The model indicates that societies that contain only small minorities of biased individuals ($f\to {0}^{+}$) may be much more prone to producing long run narratives that deviate significantly from their initially available information set (see Eq. (3)) than societies where the vast majority of the agents actively propagate biases. This resonates, for example, with Gallup survey data about vaccine beliefs in the US population, where only $6 \%$ of respondents report their belief in the relationship between vaccines and autism, but more than $50 \%$ report to be unsure about it and almost $75 \%$ report to have heard about the disadvantages of vaccinations^45^. Similarly, the model suggests that mild levels of confirmation bias ($q\ll 1$) may prove to be the most damaging in this regard, as they cause societies to live on a knife-edge where small fluctuations in the information set initially available to the biased agent population can completely censor information signals from opposing viewpoints (see Fig. 2). All in all, the model suggests that a *lack* of confirmation bias can ensure that small biased minorities much more easily hijack and dictate public discourse.

The model suggests that as the prevalence of biased agents grows, the available balance of information improves and society is more likely to maintain a long term narrative that is representative of all the information available. On the other hand, it suggests that such societies may grow more polarised. When we examine the net effect of this trade off between bias and polarization through an ensemble approach, our model suggests that the *expected* accuracy of a society may initially improve with the growth of confirmation bias, then reach a maximum at a value ${f}^{\ast }$ before marginal returns to confirmation bias are negative, i.e. confirmation bias experiences an “optimal” intermediate value. The model suggests that such value and its corresponding accuracy should increase monotonically with a society’s connectivity, meaning that more densely connected societies can support a greater amount of biased reasoners (and healthy debate between biased camps) before partitioning into echo chambers and suffering from polarization.

## Supplementary information

Supplementary Information file.

## Data Availability

The data used to test the model’s predictions on global warming beliefs were gathered from the Yale Programme on Climate Change Communication 2016 Opinion Maps^42^, which provides state and county level survey data on opinions on global warming, as well as behaviours such as the propensity to discuss climate change with friends and family. These data were combined with FCC 2016 county level data on residential high speed Internet access (www.fcc.gov/general/form-477-county-data-internet-access-services). Also, a supplemental source was used in the data aggregated by the Joint Economic Council’s Social Capital Project www.jec.senate.gov/public/index.cfm/republicans/socialcapitalproject), a government initiative aiming to measure social capital at a county level by aggregating a combination of state and county level data from sources such as the American Community Survey, the Current Population Survey, and the IRS.
