# Supplementary material for: A minimalistic model of bias, polarization and misinformation in social networks
Source: Sci Rep. 2020 Mar 26;10:5493. doi: 10.1038/s41598-020-62085-w (PMC7099021; doi:10.1038/s41598-020-62085-w)
Supplement: Supplementary file 1 — Supplementary Information file. [file 41598_2020_62085_MOESM1_ESM.pdf]

Supplementary Materials for
A minimalistic model of bias, polarization and misinformation in social networks

Orowa Sikder,[1] Robert E Smith,[1] Pierpaolo Vivo,[2] Giacomo Livan[1,3*]

[1]Department of Computer Science, University College London, London WC1E 6EA, United Kingdom
[2]Department of Mathematics, King's College London, Strand, London WC2R 2LS, United Kingdom
[3]Systemic Risk Centre, London School of Economics and Political Sciences, London WC2A 2AE, United Kingdom

*To whom correspondence should be addressed; E-mail: g.livan@ucl.ac.uk.

The PDF file includes:

- Section S1: Proof of correspondence between update dynamics and a DeGroot update process
- Section S2: Convergence proofs
- Section S3: Proof that long run steady state under stochastic updates converges almost surely to long run steady state under deterministic updates
- Section S4: Mean field calculations and numerical simulation results of signal mix distribution
- Section S5: Results on accuracy and and numerical simulation results of accuracy predictions
- Section S6: Regression results

- Figure S1: Illustration of two-node symmetric network and ghost node formulation
- Figure S2: Illustration of non-convergent behaviour in two-node dynamics
- Figure S3: Illustration of star network
- Figure S4: Illustration of neighbourhood size and covariance decay
- Figure S5: Numerical simulations of non-monotonic accuracy
- Table S1. Initial regression results between global warming beliefs, social discussion and internet access
- Table S2. IV regression results between global warming beliefs, social discussion and internet access

1 Section S1: Update dynamics

1.1 Update dynamics as random matrix $A(t)$.

Consider the set of signals $s_i(t)$ possessed by a positively oriented agent i at time t (i.e., $i \in \mathcal{B}^+$). This will consist of a set of signals retained from the previous time step, $s_i(t-1)$, and a set of biased signals $s'_i(t)$ constructed from the signals available from the nodes $j \in \partial_i$ at the end of time $(t-1)$.

Let $s^*_i(t) = \bigcup_j s_j(t-1)$ be the set of the unbiased signals available to node i at time t, i.e. the set of nodes i will receive from her neighbors before applying the confirmation bias function. Let $s_i^{*(a)}$ ($a = 1, \dots, k(k+1)^{t-1}$) be a generic signal in the set $s^*_i(t)$. After the application of the confirmation bias function, this will be turned into a signal $s_i^{\prime(a)}(t) \in s'_i(t)$ such that $s_i^{\prime(a)}(t) = \pm s_i^{*(a)}(t)$ according to the following probabilities:

$$\begin{aligned}
\mathrm{Prob}(s_i'^{(a)} = +1 | s_i^{*(a)} = -1) &= q \\
\mathrm{Prob}(s_i'^{(a)} = -1 | s_i^{*(a)} = -1) &= (1-q) \\
\mathrm{Prob}(s_i'^{(a)} = +1 | s_i^{*(a)} = +1) &= 1 \\
\mathrm{Prob}(s_i'^{(a)} = -1 | s_i^{*(a)} = +1) &= 0 \, .
\end{aligned}$$

According to the above rules, agent i checks the value of the new incoming signal, and flips it with probability q if it is incongruent with respect to her current orientation. This is entirely equivalent to node i sampling with probability q from the set $s_i^*(t)$, and with probability $1-q$ from an equally sized set of positive signals belonging to a positively oriented "ghost" node.

Let us consider the number $N_i^+(t)$ of positive signals possessed by agent i at time t. Due to the above rules, its time evolution will be such that

$$N_i^+(t) = N_i^+(t-1) + \sum_{j \in \partial_i} \left(N_j^+(t-1) + w_i(t) N_j^-(t-1) \right) \ ,$$

where $w_i(t) \in [0,1]$ is a random variable denoting the fraction of negative signals successfully distorted by i of those received by its neighbours at time t, with distribution such that $w_i(t) N_{\partial_i}^-(t) \sim \mathrm{Bin}(N_{\partial_i}^-(t), q)$, where $N_{\partial_i}^-(t)$ is the number of negative signals received by i from her neighbourhood at time t. When considering agent i's signal mix[1], the above translates to

$$x_i(t) = \frac{1}{k+1} \left(x_i(t-1) + (1 - w_i(t)) \sum_{j \in \partial_i} x_j(t-1) + w_i(t) k \right) \ . \tag{1}$$

Similarly, for a negatively oriented biased agent (i.e., $i \in \mathcal{B}^-$) we have

$$x_i(t) = \frac{1}{k+1} \left(x_i(t-1) + (1 - w_i(t)) \sum_{j \in \partial_i} x_j(t-1) \right) \ , \tag{2}$$

[1] We recall that the signal mix, as per Eq. (1) of the main paper, is defined as the fraction of positive signals possessed by an agent at a certain time, i.e., $x_i(t) = N_i^+(t)/(N_i^+(t) + N_i^-(t)) = N_i^+(t)/(k+1)^t$.

with $w_i(t)N^+_{\partial_i}(t) \sim \mathrm{Bin}(N^+_{\partial_i}(t), q)$.

Combining Eqs. (1) and (2) with the time evolution for the signal mix of unbiased agents, which reads

$$x_i(t) = \frac{1}{k+1}\left(x_i(t-1) + \sum_{j \in \partial_i} x_j(t-1)\right) ,$$

we can see that the time evolution for the vector of signal mixes $x(t)$ can be written as

$$\hat{\boldsymbol{x}}(t) = \hat{A}(t)\hat{\boldsymbol{x}}(t-1) , \tag{3}$$

where $\hat{\boldsymbol{x}} = [\boldsymbol{x}^T, 1, 0]^T$ where the latter terms represent the (fixed) signal mixes of the ghost nodes and the $\hat{\boldsymbol{x}}(t)$ the signal mixes of the original set. $\hat{A}(t)$ is an $(n+2) \times (n+2)$ random matrix with entries with a block structure as follows:

$$\hat{A}(t) = \left[\begin{array}{c|c} Q(t) & R(t) \\ \hline 0 & I \end{array}\right] ,$$

where $Q(t)$ is an $(n \times n)$ matrix representing the original graph structure with $Q_{ii}(t) = \frac{1}{k+1}$, $Q_{ij}(t) = \frac{1}{k+1}$ where i is a unbiased agent connected to j, $Q_{ij}(t) = \frac{1-w_i(t)}{k+1}$ where i is a biased agent connected to j, and 0 otherwise. $R(t)$ is an $(n \times 2)$ matrix representing connections from biased agents to their preferred ghost node (which we index by $+$ and $-$). $R_{i+}(t) = \frac{w_i(t)k}{k+1}$ if $i \in \mathcal{B}^+$ and 0 otherwise. Analogous weights exist for negatively biased agents to the negative ghost node. I is a (2×2) identity matrix representing the weights of ghost nodes to themselves. 0 is the $(2 \times n)$ block of zeros representing the (lack of) edges outbound from the ghost nodes.

Finally, it is worth noting that the above formulation consisting of two ghost nodes is fully equivalent to a formulation where each biased agent has a "personalized" ghost node that reflects their positive or negative orientation appropriately. In this case $\hat{A}(t)$ is an $(n + fn) \times (n + fn)$ matrix with an extra fn ghost nodes added, one for each biased agent. However, while this formulation has a more favourable interpretation in terms of "content personalization", it is less convenient analytically, so for the reminder of the Supplementary Information the simplified ghost node formulation will be utilised.

1.2 Almost sure convergence of $\hat{A}(t)$.

We now proceed to show that stochastic weights $w_i(t)$ appearing in the matrix $\hat{A}(t)$ of (3) converge almost surely to q when $t \to \infty$ as long as at least one signal of each type is held by at least one node in the network. As such the random matrix $\hat{A}(t)$ converges almost surely to a fixed matrix $\hat{A} = \mathbb{E}(\hat{A}(t))$.

Let us consider $i \in \mathcal{B}^+$. As established in the previous section, $w_i(t)$ is simply the fraction of negative signals held by node i's neighbours that i successfully flips to positive at time t. Let us also recall that $N^-_{\partial_i}(t)$ represents this set of negative signals available from all $j \in \partial_i$, and that each one is independently flipped to positive with probability q. If we can establish that $N^-_{\partial_i}(t)$ grows indefinitely as $t \to \infty$, the Strong Law of Large Numbers (SLLN) can then be invoked to establish the desired result. Since $N^-_{\partial_i}(t) = \sum_{j\in\partial_i} N^-_j(t)$, then if i's neighbours possess an increasing and unbounded number of negative signals over time, then $N^-_{\partial_i}(t)$ will also be increasing and unbounded. As such, each $w_i(t)$ will converge almost surely to q.

Consider an arbitrary $j \in \partial_i$. Note that since information sets are retained by agents at every time step, we can immediately rule out the possibility of that the number of negative signals held by agent j shrinks over time, and we merely need to show that her set of negative signals does not remain constant over time.

Let us assume that at least one negative signal has been injected into the network at $t = 0$, and that one agent ℓ possesses such negative signal. In a strongly connected network (such as the k-regular network we consider in the main paper), there exists at least one directed path from k to j of length d. Let us indicate the probability of a negative signal successfully being transmitted from an agent a to an agent b along such path as p_{ab}. We note that $p_{ab} = 1 - q$ if $b \in \mathcal{B}^+$ and $p_{ab} = 1$ otherwise. Therefore, the probability of the signal successfully reaching j in d time steps is:

$$p_{\ell j} = \prod_{(a,b)} p_{ab} \geq (1-q)^d > 0 \ ,$$

Which allows us to conclude that at each time step $t > d$ there exists a strictly positive probability that a negative signal is added to j's information set. This, in turn, implies that the set of negative signals obtained by j will grow without bound for $t \to \infty$, which establishes our result. Since this occurs for each w_i, we can conclude also that $\hat{A}(t) \xrightarrow{a.s.} \hat{A}$, as well as the block submatrices $Q(t) \xrightarrow{a.s.} Q$ and $R(t) \xrightarrow{a.s.} R$. The edges of these fixed matrices are identical to the structure outlined in the previous section except w_i is replaced with q.

Finally it is worth noting that this convergence result depends only on the strong connectedness of $\mathcal{G}$ and not on the edges from the biased agents to the ghost nodes. This is important as this means that even as the orientations of the biased agents change (which is reflected in the rewiring of these ghost node edges), the almost sure convergence is not interrupted.

2 Section S2: Biased agents settle in their orientation

In this section, we show that biased agents cannot continue to switch orientation indefinitely, and instead settle into a fixed set of orientations given sufficient time. Recall that a biased agent $i \in \mathcal{B}$ switches her orientation $y_i(t)$ when her information sets switches from a majority of positive signals ($x_i(t) > 1/2$) to a majority of negative signals ($x_i(t) < 1/2$), or vice versa.

We begin by arguing that in some network topologies there exists some t after which biased agents cease switching their orientation. For convenience, we define a network as *settled* at t^* if for all $t > t^*$, $y_i(t^*) = y_i(t)$ for all $i \in \mathcal{B}$. To do this, we first consider an "adversarial" toy example designed to maximise the likelihood of indefinite switching, and show that assuming perpetual switching leads to a contradiction even in this case. We then go on to show how other, more complex, network topologies are also guaranteed to settle. We limit to two topologies for brevity but these results can be extended. Alongside the extensive evidence from numerical simulations, we argue that the model is likely to settle for any arbitrary graph.

2.1 Two node network.

Consider a network with two nodes, labeled 1 and 2 respectively, both of which are biased agents. Each node has a self-weight of y and a weight of $(1-y)$ on its sole neighbour[2]. This schematic is illustrated in Figure S1. As has been established in 1, the signal distortion dyanmics can be mimicked by introducing two ghost nodes that represent a source of positive and negative signals respectively. The weights associated with these ghost nodes are random variables that converge almost surely to q as $t \to \infty$.

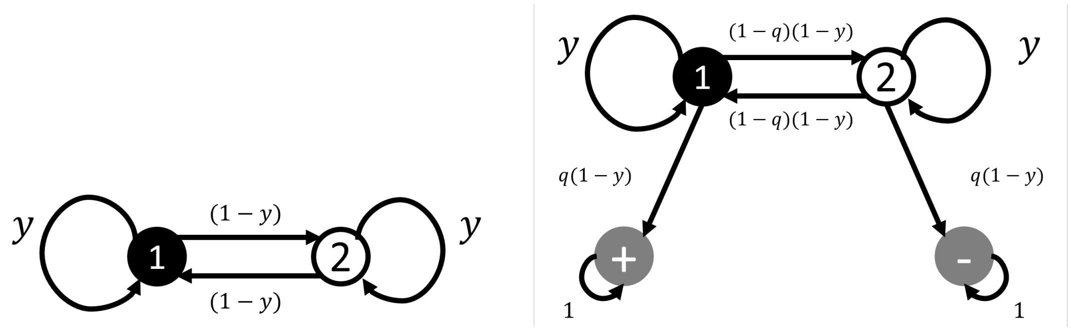

Figure S1: Left: A schematic of the two node symmetric network. Right: A schematic of the two node symmetric network where ghost nodes are introduced to mimic the effect of the biased signals.

In what follows, we show that this simplified model settles (i.e., both biased agents settle at a finite time on a pair of orientations that they do not thereafter change). For the purposes of illustration, for the moment let us consider the asymptotic case where the random weights have converged to a deterministic set of weights (q).

The outline of this proof (and subsequent ones on alternative network structures) is to establish that in order for a biased agent i to switch orientation, their neighbours must have signal mixes sufficiently far from i's that they can cause i to switch orientation despite the fact that i's ghost node biases her learning to maintain "inertia" in the current orientation. However, at the same time, the network structure ensures that nodes tend to converge closely to their neighbourhood, which eventually prevents switching from occurring.

[2]This setting generalizes the one introduced in Eqs. (1) and (2), which is recovered for $k = 1$ and $y = 1/2$.

The proof follows by contradiction. Suppose that the model never stabilizes, i.e., that at least one of the biased agents keeps switching perpetually. Suppose node 1 switches at arbitrary times $\{T\} = \ldots < t_{n-2} < t_{n-1} < t_n < \ldots$. We do not assume for now that times in T are over consecutive time steps, the gap between them can be as large as intended (see Fig. S2).

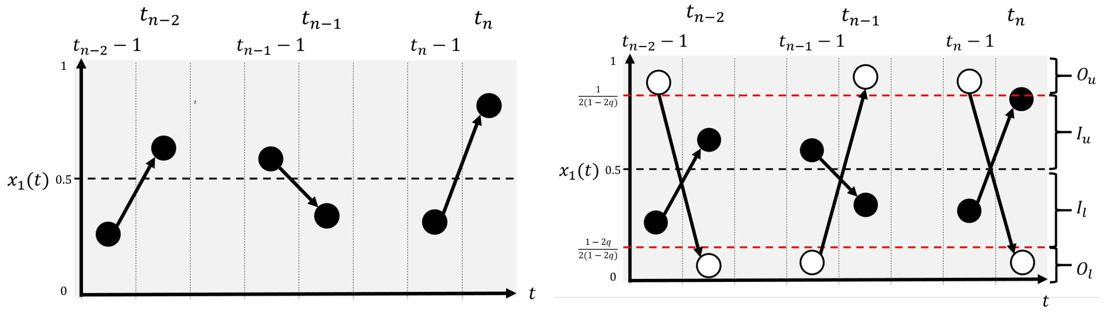

Figure S2: By assumption, node 1 continues to switch orientation at arbitrary time steps t_{n-2}, t_{n-1}, t_n by crossing the threshold signal mix $x_i = \frac{1}{2}$. The threshold is denoted by a dashed line. If node 1 switches, its neighbour (node 2, white) must cross sufficiently distant thresholds, denoted by the red dashed lines. Furthermore, the switches must be simultaneous, or else the switching terminates perpetually. The regions $\mathcal{O}_u, \mathcal{I}_u, \mathcal{I}_l, \mathcal{O}_l$ are outlined.

Consider some arbitrary t_n, where x_1 switches from $x_1(t_n - 1) < 1/2$ to $x_1(t_n) > 1/2$. Using the model's update rule (see (2)) we can note:

$$x_1(t_n) = yx_1(t_n - 1) + (1-q)(1-y)x_2(t_n - 1) > \frac{1}{2}\,, \tag{4}$$

and using the fact that $x_1(t_n - 1) < 1/2$ we get ,

$$\frac{y}{2} + (1-q)(1-y)x_2(t_n - 1) \geq \frac{1}{2}$$

which in turn implies

$$x_2(t_n - 1) \geq \frac{1}{2(1-q)} > \frac{1}{2}\,.$$

By following the same reasoning one can show that an x_1 switch in the opposite direction would imply

$$x_2(t_n - 1) \leq \frac{1-2q}{2(1-q)} = \frac{1}{2} - \frac{q}{2(1-q)} < \frac{1}{2}\,.$$

Therefore, for node 1 to switch endlessly, then node 2 must also do so, and cannot start from an arbitrary point, but rather has to either be above $1/(2(1-q))$ or below $(1-2q)/(2(1-q)$ at time $t_n - 1$ for x_1 to cross the $1/2$ line at time t_n from below or above, respectively. For the sake of convenience we introduce the following regions

$$\begin{aligned}
\mathcal{O}_l &= \left[0, \frac{1-2q}{2(1-q)}\right) \\
\mathcal{I}_l &= \left[\frac{1-2q}{2(1-q)}, \frac{1}{2}\right) \\
\mathcal{I}_u &= \left[\frac{1}{2}, \frac{1}{2(1-q)}\right) \\
\mathcal{O}_u &= \left(\frac{1}{2(1-q)}, 1\right] ,
\end{aligned}$$

Where the subscripts indicate whether the interval lies in the upper or lower hemisphere (above and below $1/2$, denoted by the subscripts u and l). We also denote the "inner" region $\mathcal{I} = \mathcal{I}_l \cup \mathcal{I}_u$ and the "outer" region $\mathcal{O} = [0,1]/\mathcal{I}$ defined by the above boundaries.

According to the above considerations, for node 1 to switch orientation to negative at t_n, then $x_2(t_n - 1) \in \mathcal{O}_l$, and for node 1 to switch to positive at t_n, then $x_2(t_n - 1) \in \mathcal{O}_u$. These regions are highlighted in S2. Clearly, the size of $\mathcal{I}$ grows with q (and $\mathcal{O}$ shrinks with q). It is worth noting that for $q > 1/2$, the inner region's boundaries exceed $[0,1]$, i.e., orientation switches are impossible. The intuition behind this is that if a node is able to flip more than half of the incongruent signals coming its way, it will never include enough incogruent signals in her information set to switch orientation.

We further note that if a node and its neighbour are ever in the same orientation, then any future switches are impossible. Indeed, if two nodes share the same orientation, they are both linked to the same ghost node. As such, the set of available signals for each node is only its neighbour and its ghost node. Regardless of the value of q, there is no way for either node to accumulate sufficient incongruent signals to switch orientation. All in all, it follows that both node 1 and node 2 must switch at the same time step whenever a switch occurs. This result is illustrated in S2.

We now show that if x_2 lies in the outer region $\mathcal{O}$, it will converge to the inner region $\mathcal{I}$. Furthermore, once it enters the inner region, it cannot leave it. Also, this ceases the switching of the node 1, since its switching requires x_2 to alternate between the upper and lower hemispheres of the outer region.

As proved above, at any given time step node 1 and its neighbour 2 can either both switch orientation, or both maintain their current orientation. We will consider both possibilities. Assume the former first, in which case we can show the two nodes must grow closer together. Suppose that at time t_n, $x_1(t_n) > 1/2$, and $x_2(t_n) < 1/2$. At $t_n + 1$, this orientation switches so $x_2(t_n + 1) > x_1(t_n + 1)$. Making use of Eqs. (1) and (2), we can write

$$\begin{aligned} x_2(t_n + 1) - x_1(t_n + 1) &= [(1 - y)(1 - q) - y]\,(x_1(t_n) - x_2(t_n)) - (1 - y)q \\ &< \delta(x_1(t) - x_2(t))\ , \end{aligned} \tag{5}$$

where $\delta = [(1 - y)(1 - q) - y] < 1$. Therefore, when the node switches orientation with their neighbour, they must converge strictly closer.[3]

We now consider the logical disjunct. Suppose instead that a switch does not occur, and at times t_n, $t_n + 1$ we have $x_1(t_n), x_1(t_n + 1) > 1/2$, and $x_2(t_n), x_2(t_n + 1) < 1/2$. Therefore, we can write

$$x_1(t_n + 1) - x_2(t_n + 1) = [y - (1 - y)(1 - q))]\,(x_1(t_n) - x_2(t_n)) + (1 - y)q.$$

If the two nodes are to move closer in this time step, then we must have $x_1(t_n+1) - x_2(t_n+1) < (1 - \mu)(x_1(t_n) - x_2(t_n))$ for some $\mu \in (0, 1)$. Using this in (2.1) we obtain the following sufficient condition for convergence:

$$x_1(t_n) - x_2(t_n) > \frac{q}{2 - q - \frac{\mu}{1-y}}\ . \tag{6}$$

[3] We require $\delta < 1$ and not $|\delta| < 1$. While $\delta < -1$ would violate the convergence criterion, it would also imply $x_2(t + 1) < x_1(t + 1)$, leading to a contradiction.

Finally, note that if $x_1(t_n) > 1/2$ and $x_2(t_n) \in \mathcal{O}_l$, then:

$$x_1(t_n) - x_2(t_n) > \frac{1}{2} - \frac{1-2q}{2(1-q)} > \frac{q}{2 - q - \frac{\mu}{1-y}} \tag{7}$$

for an arbitrarily small μ. Thus, if $x_2(t_n) \in \mathcal{O}_l$, then the two nodes are sufficiently far apart that the condition in (6) holds, and the two nodes must converge closer together. The parallel argument can be made for the opposite starting orientations.

Even if a switch does not occur, then the nodes will converge strictly closer. Indeed, We have established that if $x_2(t) \in \mathcal{O}$, then at each time step the distance $|x_1(t) - x_2(t)|$ must strictly shrink. As such, the nodes will eventually become close enough that $x_2(t) \in \mathcal{I}$, and switching of node 1 ceases.

We complete the proof by showing that once node 2's signal mix has entered the inner region $\mathcal{I}$, it cannot leave it. We have established already that nodes must have opposing orientations at all times. Let us consider the case where $x_2(t_n) \in \mathcal{I}_u$ and $x_1(t_n) < 1/2$. Suppose by contradiction that in time step $t_n + 1$ node 2 is able to "escape" $\mathcal{I}$ from below, going from below $1/(2(1-q))$ to above such value (i.e., to $\mathcal{O}_u$). This implies

$$\begin{aligned} \frac{1}{2(1-q)} &< x_2(t_n+1) = yx_2(t_n) + (1-y)(1-q)x_1(t_n) + (1-y)q \\ &< \frac{y}{2(1-q)} + \frac{1}{2}(1-y)(1-q) + (1-y)q \ , \end{aligned}$$

which leads to $1 + q < (1-q)^{-1}$, i.e. to the impossible result $q^2 < 0$. Therefore, node 2 cannot go from $\mathcal{I}$ to $\mathcal{O}_u$. Finally we also know that it cannot go from $\mathcal{I}$ to $\mathcal{O}_l$ as this would require both nodes to switch orientation, which is ruled out because $x_2(t) \in \mathcal{I}$. A parallel argument can be made if the orientations are reversed. Thus, the two node symmetric network will always converge to a region of the signal mix space where the nodes' signal mixes are too close to support any switch of orientation, arriving at the desired result.

The above proof can be easily replicated after relaxing the simplifying asymptotic assumption that q is fixed. This can be done by reintroducing the time-dependent random weights $w_i(t)$

($i = 1, 2$), and recalling that, due to their almost sure convergence to q, for any $\epsilon > 0$ there exists a time t^* such that for all $t > t^*$ and for all i

$$q - \epsilon < w_i(t) < q + \epsilon \ .$$

Adjusting the bounds used in the convergence proof to include the above time evolution allows to obtain the same result.

2.2 Star network.

Let us now consider a k-star network of biased agents, with the central node labeled as 0 and branch nodes labeled as $1, \ldots, k$[4]. As before, allow y to be the self-weight of each node and q the confirmation bias parameter. Assume for simplicity that the central node has a weight of $(1 - y)/k$ on each branch node.

Firstly, note that if any branch node switches indefinitely, then the central node 0 must also switch indefinitely (or else there would be no "driving force" causing the branch nodes to switch). So, let us focus on showing that it is impossible for the central node to do so. The logic of the two-node network proof can be followed almost exactly by replacing $x_1(t)$ and $x_2(t)$ with $x_0(t)$ and $\sum_{j=1}^{k} x_j(t)/k$, respectively.

The first set of results up to (2.1) follow precisely given the substitution of terms above. We use this to establish once again that for $x_0(t)$ to switch indefinitely $\sum_{j=1}^{k} x_j(t)/k$ must oscillate between $1/(2(1 - q))$ and $(1 - 2q)/(2(1 - q))$, i.e., between the upper and lower hemispheres of the outer region $\mathcal{O}$. Furthermore, whenever the central node switches orientation, the branch nodes' average signal mix must also change from above to below $1/2$ (or vice versa), even if none of the branch nodes in particular switch orientation.

[4]Strictly speaking, the signal diffusion mechanism would need to be modified for non-regular graphs to allow for signal diffusion to be equivalent to node averaging. More complex regular structures can also be shown to converge, but a star graph permits us to show how convergence holds even with a strikingly different topology. We proceed with the star graph for the purpose of illustration.

The next steps follow closely those of the two-nodes network. Suppose firstly that the central node switches orientation (and the branch nodes' average must also shift accordingly). Suppose that at time t_n, $x_0(t_n) > 1/2$, and $\sum_{j=1}^{k} x_j(t_n)/k < 1/2$. At $t_n + 1$, this orientation switches so that $\sum_{j=1}^{k} x_j(t_n + 1)/k > x_0(t_n + 1)$. Adapting Eqs. (1) and (2) to the present case, we have

$$\begin{aligned}\frac{1}{k}\sum_{j=1}^{k} x_j(t_n+1) - x_0(t_n+1) &= \left[\frac{1}{k}\sum_{j=1}^{k}\left(x_j(t_n) + (1-y)(1-q)x_0(t_n) + (1-y)qg_j(t_n)\right)\right] \\ &- \left[yx_0(t_n) + (1-y)(1-q)\frac{1}{k}\sum_{j=1}^{k} x_j(t_n) + (1-y)q\right] ,\end{aligned}$$

where we have introduced a new indicator variable such that $g_j(t) = 1$ is node j is positively oriented at time t, and $g_j(t) = 0$ otherwise. Let $g(t) = \sum_{j=1}^{k} g_j(t)/k$ be the fraction of positively oriented branch nodes. We can then simplify the above:

$$\begin{aligned}\frac{1}{k}\sum_{j=1}^{k} x_j(t_n+1) - x_0(t_n+1) &= [(1-y)(1-q)-y]\left(x_0(t_n) - \frac{1}{k}\sum_{j=1}^{k} x_j(t_n)\right) - (1-y)q(1-g(t_n)) \\ &< [(1-y)(1-q)-y]\left(x_0(t_n) - \frac{1}{k}\sum_{j=1}^{k} x_j(t_n)\right) ,\end{aligned}$$

where we used the fact that $(1-y)q(1-g(t_n)) > 0$. This can be rewritten as

$$\frac{1}{k}\sum_{j=1}^{k} x_j(t_n+1) - x_0(t+1) < \delta\left(x_0(t_n) - \frac{1}{k}\sum_{j=1}^{k} x_j(t_n)\right) ,$$

where $\delta = [(1-y)(1-q)-y] < 1$, which re-establishes the result of (5): if the central node flips, it must converge strictly closer to the branch nodes. Next, we establish that in the time steps where the central node does not switch orientation, the centre and branches still converge as long as the branch average is within $\mathcal{O}$. The reasoning follows the one of the previous section exactly given the appropriate substitutions, and we can replace the condition in (6) with:

$$x_0(t_n) - \frac{1}{k}\sum_{j=1}^{k} x_j(t) > \frac{q(1-g(t_n))}{2-q-\frac{\mu}{1-y}} . \tag{8}$$

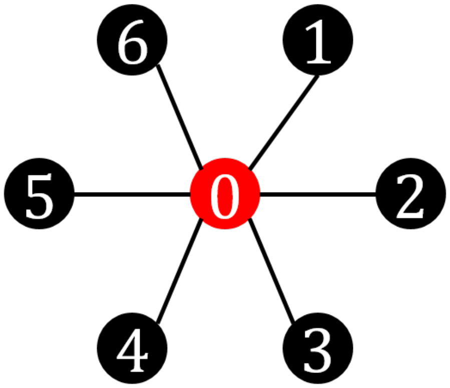

Figure S3: A network structure consisting of a central node 0 and $k = 6$ branch nodes. All nodes are biased agents for the purposes of the toy example.

Recall that $x_0(t_n) > 1/2$ and $\sum_{j=1}^{k} x_j(t)/k < (1-2q)(2(1-q)) \in \mathcal{O}_l$, therefore:

$$x_0(t_n) - \frac{1}{k}\sum_{j=1}^{k} x_j(t) > \frac{1}{2} - \frac{1-2q}{2(1-q)} > \frac{q}{2-q-\frac{\mu}{1-y}} \geq \frac{q(1-g(t))}{2-q-\frac{\mu}{1-y}}$$

for an arbitrarily small $\mu > 0$. Hence, even if a switch does not occur, then the nodes will converge strictly closer.

The final steps of the proof mirror those that follow (7) of the previous section, except a factor of $g(t_n)$ dampens the ability of the branch nodes to escape the inner region even further. As such, we establish that even on a star network structure, the biased agents cannot switch their orientation endlessly, and must eventually converge.

2.3 Simulated dynamics and convergence criteria.

As has been established in the previous sections, settling is guaranteed under some simple network topologies chosen specifically to hinder convergence. We round out the argument by noting that settling also occurs in simulations for the k-regular network employed throughout the paper and in the following proofs.

In what follows, we establish criteria for the case of a fixed q. Analogous criteria can be easily established for the case of stochastic convergent weights $w_i(t)$ instead, although without

much adding much insight. Furthermore, in practice the stochasticity rapidly settles in numerical simulations, meaning that convergence can be safely studied using the asymptotic fixed q assumption.

In order to guarantee that a network has in fact settled over the course of a simulation, we identify a "settling" rule for the signal mix $\hat{\boldsymbol{x}}(t)$. As we demonstrate in the following section, if one assumes that the biased agents at time t no longer switch orientations, one can calculate the steady state that would arise from this configuration of biased agents. Call this $\hat{\boldsymbol{x}}^*(\hat{\boldsymbol{x}}(t))$. We can show that if the signal mix $\hat{\boldsymbol{x}}(t)$ is sufficiently close to its corresponding steady state $\hat{\boldsymbol{x}}^*(\hat{\boldsymbol{x}}(t))$ it will converge uniformly to that steady state without any further changes to any agent's orientation.

Define the difference between a signal mix and its steady state:

$$\epsilon(t) = \hat{\boldsymbol{x}}(t) - \hat{\boldsymbol{x}}^* \ .$$

Recalling that the model's dynamics is such that

$$\hat{\boldsymbol{x}}(t) = \hat{A}\hat{\boldsymbol{x}}(t-1)$$

we then have

$$\hat{\boldsymbol{x}}^* + \epsilon(t) = \hat{A}\hat{\boldsymbol{x}}^* + \hat{A}\epsilon(t-1) = \hat{\boldsymbol{x}}^* + \hat{A}\epsilon(t-1) \ ,$$

and therefore:

$$\epsilon(t) = \hat{A}\epsilon(t-1) \ .$$

Finally, define $\epsilon^*(t) = \max_i(|\epsilon_i(t)|) = ||\epsilon(t)||_\infty$. Then for any arbitrary biased node:

$$\epsilon_i(t+1) = \hat{a}_{ii}\epsilon_i(t) + (1-q)\sum_j \hat{a}_{ij}\epsilon_j(t) \ ,$$

where $\hat{a}_{ij}$ is the weight between node i and j in matrix $\hat{A}$, and we use the fact that the ghost nodes are always at their exact steady state, so their $\epsilon_G = 0$. Then taking the absolute distance and using the triangular inequality:

$$|\epsilon_i(t+1)| \leq \hat{a}_{ii}|\epsilon_i(t)| + (1-q)\sum_j \hat{a}_{ij}|\epsilon_j(t)|$$
$$\leq \hat{a}_{ii}|\epsilon^*(t)| + (1-q)\sum_j \hat{a}_{ij}|\epsilon^*(t)| = (1-q(1-\hat{a}_{ii}))|\epsilon^*(t)| < |\epsilon^*(t)| \; ,$$

and similarly for an unbiased agent, we can show:

$$|\epsilon_i(t+1)| \leq |\epsilon^*(t)| \; .$$

In short, for each steady state once the current signal mixes are within some ϵ-cube of the steady state, they must remain within that ϵ-cube. Furthermore, biased agents at each time step must converge strictly closer to the steady state. A larger q or smaller self-weight ($\hat{a}_{ii}$) will cause faster convergence.

Finally, we can also note because the network is strongly connected, there are some $r \geq 1$ steps between each unbiased and a biased node, and so it can be shown that in a finite number of steps *all* nodes must converge strictly closer to the steady state than the maximum threshold of the ϵ-cube.

Given all the above, we can now explicitly outline a "stable" region. Denote:

$$\epsilon_s = \min_i \left(|x_i^* - \frac{1}{2}| \right) \; .$$

That is, the closest any of the steady states are to the threshold. If the current signal mixes can get within the ϵ_s-ball of the steady state, then there can be no crossing the 0.5 threshold and as such the steady state cannot move - this is a sufficient condition for settling to be guaranteed.

Using this condition, we are able to demonstrate settling occurs for a wide range of parameters for k-regular networks. We tested the condition on 1000 iterations each of the following parameter sets: $n = 10^3$, $p = 0.5$, $k = \{3, 4, 5, 10, 100, 999\}$, $f = \{.05, .1, .2, .5, 1\}$, $q = \{.05, .1, .25, .5, .75.1\}$. The settling criteria was successfully reached for every single run of the model, establishing extremely high confidence that the k-regular biased information aggregation model always settles.

3 Section S3: Convergence of signal mixes

So far, we have established that the random update matrix $A(t)$ converges almost surely to a fixed update matrix A. Furthermore, we have demonstrated with extremely high confidence that biased agents settle in their orientation after some finite time. As such, for biased k-regular networks, assume that there exists some time t^* after which biased agents cease switching their orientation. Define $\hat{y}^*_{\mathcal{B}}$ as the steady state fraction of positively oriented biased agents. Then the following holds.

(1) The signal mix vector $\hat{\boldsymbol{x}}(t)$ converges to some $\hat{\boldsymbol{x}}^* = \hat{A}^*\hat{\boldsymbol{x}}(0)$ for both biased and unbiased networks, where $\hat{A}^*$ is a steady-state matrix of influence weights which can be computed explicitly.

(2) Unbiased networks achieve consensus, and converge to influence weights of $a^*_{ij} = 1/n$ for all pairs (i, j). This ensures that, for all $i \in V$, $x^*_i = x^*_V = \bar{x}(0)$, where $\bar{x}(0) = \sum_{i=1}^{n} s_i$ is the intial average signal mix.

(3) Biased networks where $\hat{y}^*_{\mathcal{B}} = 0, 1$ achieve consensus, and converge to influence weights $\hat{a}^*_{ij} = 0$ for all pairs $(i, j) \in V$, $\hat{a}^*_{i+} = \hat{y}^*_{\mathcal{B}}$ and $\hat{a}^*_{i-} = 1 - \hat{y}^*_{\mathcal{B}}$ for all $i \in V$.

(4) Biased networks where $0 < \hat{y}^*_{\mathcal{B}} < 1$ do not achieve consensus, and converge to influence weights $\hat{a}^*_{ij} = 0$ for all $(i, j) \in V$, and $\hat{a}^*_{i+} + \hat{a}^*_{i-} = 1$ for all $i \in V$.

We note that results regarding unbiased networks (part of (1) and all of (2)) are already well established results (see, for example, [1]) and are listed purely for comparison with biased networks. We focus on proving the remainder of the results.

The results follow from the structure of $\hat{\boldsymbol{x}}^* = \hat{A}^*\hat{\boldsymbol{x}}(0) = \lim_{t\to\infty} \prod_{\tau=0}^{t} \hat{A}(\tau)\hat{\boldsymbol{x}}(0)$. We proceed by demonstrating that despite the stochasticity in the random update mechanism $\hat{A}(\tau)$, the steady state converges to a fixed vector $\hat{\boldsymbol{x}}^*$.

First note that for $\tau > t^*$ the biased agents will have ceased switching their orientation, and the random update matrix $\hat{A}(\tau)$ will have a fixed underlying structure $\hat{A} = \mathbb{E}(\hat{A}(\tau))$. The proof will follow by demonstrating that $\lim_{t\to\infty} \prod_{\tau=0}^{t} \hat{A}(\tau) = \lim_{t\to\infty} \hat{A}^t$. That is, the products of random matrices converges to the products of their expectation.

Firstly recall the block structure of $\hat{A}(\tau)$:

$$\hat{A}(\tau) = \left[\begin{array}{c|c} Q(\tau) & R(\tau) \\ \hline 0 & I \end{array}\right] ,$$

with dimensions (clockwise from top-left): $(n \times n)$, $(n \times 2)$, (2×2), (2×2). Important properties of the blocks include:

$$Q(\tau) = Q + \epsilon_Q(\tau) \xrightarrow{a.s.} Q$$
$$R(\tau) = R + \epsilon_R(\tau) \xrightarrow{a.s.} R .$$

The properties above indicate that the blocks converge to their deterministic counterparts almost surely. This allows us to state that for any ϵ and matrix norm $||.||$, there is guaranteed some $t' \geq t^*$ such that for all $\tau > t'$, $||Q - Q(\tau)|| = ||\epsilon_Q(\tau)|| < \epsilon$. Also, the matrix Q is such that

$$\sum_j Q_{ij} < 1 \;\; \forall i \in \mathcal{B}$$
$$\sum_i Q_{ij} < 1 \;\; \forall j \in \partial\mathcal{B} .$$

The properties respectively indicate that the limit matrix Q is both row and column sub-stochastic. Row sub-stochasticity follows from the outgoing edges from the set of biased agents ($\mathcal{B}$). For the k-regular graphs that are the focus of our analysis this can be specifically shown to be $\frac{(1-q)k+1}{k+1}$. Column sub-stochasticity follows from the neighbours of the biased agents ($\partial\mathcal{B}$) having incoming connections necessarily less than 1.

We now define the product of the *random* matrices $\hat{A}(\tau)$ as:

$$\prod_{\tau=0}^{t} \hat{A}(\tau) = \tilde{A}(t,0) = \left[\begin{array}{c|c} \tilde{Q}(t,0) & \tilde{R}(t,0) \\ \hline 0 & I \end{array}\right] ,$$

where $\tilde{Q}(t,0$ and $\tilde{R}(t,0)$ are placeholder terms for the the random block matrix products which arise through products of the random matrices $\hat{A}(\tau)$. Consider also the deterministic analog to this expression:

$$\prod_{\tau=0}^{t} \hat{A} = \hat{A}^t = \dot{A}(t,0) = \left[\begin{array}{c|c} \dot{Q}(t,0) & \dot{R}(t,0) \\ \hline 0 & I \end{array}\right] .$$

This formulation defines a random and analogous deterministic sequence for each of the blocks, denoted by $\tilde{A}(t,0)$ and $\dot{A}(t,0)$ respectively.

Firstly, we demonstrate that $\lim_{t\to\infty} \dot{Q}(t,0) = \lim_{t\to\infty} Q^t = \mathbf{0}$. Consider the 2-norm $||.||_2$. We consider first the deterministic matrix Q. Recall that since Q is doubly sub-stochastic, Q^TQ is necessarily sub-stochastic and therefore:

$$||Q|| = (1-\delta) < 1 ,$$

for some $\delta \in (0,1)$. It follows that:

$$\lim_{t\to\infty} ||Q^t|| \leq \lim_{t\to\infty} ||Q||^t = \lim_{t\to\infty} (1-\delta)^t = 0 ,$$

therefore $\lim_{t\to\infty} Q^t = \mathbf{0}$ (making use of the fact that $||X|| = 0 \iff X = \mathbf{0}$). Now consider the term of interest $\tilde{Q}(t,0)$:

$$||\tilde{Q}(t,0)|| = ||\prod_{0}^{t} Q(\tau)|| \leq \prod_{0}^{t} ||Q(\tau)|| .$$

Note that $||Q(\tau)|| \leq 1$ for all τ. However we can show that almost all $||Q(\tau)|| < 1$:

$$||Q(\tau)|| = ||Q + \epsilon_Q(\tau)|| \leq ||Q|| + ||\epsilon_Q(\tau)|| = (1-\delta) + ||\epsilon_Q(\tau)||$$

We select some t' such that for all $\tau > t'$, $||\epsilon_Q(\tau)|| = \mu < \delta$ for some $\mu > 0$. Therefore:

$$||Q(\tau)|| \leq (1 - \delta + \mu) = (1 - \delta^*) < 1 \,, \tag{9}$$

where $\delta^* = \delta + \mu$. We can now conclude:

$$\lim_{t\to\infty} ||\tilde{Q}(t,0)|| \leq \lim_{t\to\infty} \prod_{t^*}^{t} (1-\delta^*) = \lim_{t\to\infty} (1-\delta^*)^t = 0 \,.$$

We now show that $\lim_{t\to\infty} \tilde{R}(t,0) = \lim_{t\to\infty} \dot{R}(t,0) = (I-Q)^{-1}R$. Consider firstly the deterministic sequence, which can be defined through the following iterative relationship:

$$\dot{R}(t,0) = Q\dot{R}(t-1,0) + R \,. \tag{10}$$

Note again that $\dot{R}(t,0)$ refers to the t-th term in a deterministic sequence whereas Q and R are specific block matrices. The expression (10) can be straightforwardly solved in the limit:

$$\lim_{t\to\infty} \dot{R}(t,0) = (I-Q)^{-1}R \,.$$

Consider now the random sequence, which can be defined analogously:

$$\tilde{R}(t,0) = Q(t)\tilde{R}(t-1,0) + R(t) \,. \tag{11}$$

Note here $\tilde{R}(t,0)$ refers to the t-th term in a random sequence and $Q(t)$ and $R(t)$ are random block matrices that occur at time $\tau = t$. In order to proceed we define:

$$\tilde{R}(t,0) = \dot{R}(t,0) + E(t) \,, \tag{12}$$

where here $E(t)$ is an error term capturing the difference between the terms of the deterministic and random sequences at time $\tau = t$. We substitute (12) into (11):

$$\tilde{R}(t,0) = Q(t)(\dot{R}(t-1,0) + E(t-1)) + R(t) \; . \tag{13}$$

We now substitute the definition of $Q(\tau)$ and $R(\tau)$ into (13):

$$\tilde{R}(t,0) = Q\dot{R}(t-1,0) + R + QE(t-1) + \epsilon_Q(t)(\dot{R}(t-1,0) + E(t-1)) + \epsilon_R(t) \; .$$

Note that we can substitute (10) for the two leading terms on the RHS:

$$\tilde{R}(t,0) - R(t,0) = QE(t-1) + \epsilon_Q(t)(\dot{R}(t-1,0) + E(t-1)) + \epsilon_R(t) \; .$$

We can now take the 2-norm $||.||_2$:

$$\begin{aligned} ||E(t)|| = ||\tilde{R}(t,0) - \dot{R}(t,0)|| &= ||QE(t-1) + \epsilon_Q(t)(\dot{R}(t-1,0) + E(t-1)) + \epsilon_R(t)|| \\ &\leq ||Q|| \; ||E(t-1)|| + ||\epsilon_Q(t)|| \; ||\tilde{R}(t-1)|| + ||\epsilon_R(t)|| \; . \end{aligned}$$

We can now substitute in (9) and once again make use of the fact that for any $0 < \epsilon$ we can define t' such that $||\epsilon_Q(t)||, ||\epsilon_R(t)|| < \epsilon$. We also note that $||\tilde{R}(t-1)|| < n$ (where n is the size of the network), therefore

$$||E(t)|| \leq (1-\delta)||E(t-1)|| + \epsilon(n+1) \; ,$$

and therefore for a sufficiently small ϵ, there is a corresponding t' such that for $t > t'$:

$$||E(t)|| \leq (1-\delta^*)||E(t-1)|| < ||E(t-1)|| \; .$$

Finally we get:

$$\lim_{t\to\infty} ||\tilde{R}(t,0) - \dot{R}(t,0)|| = \lim_{t\to\infty} ||E(t)|| = 0 \ ,$$

which allows us to conclude that $\lim_{t\to\infty} \tilde{R}(t,0) = \lim_{t\to\infty} \dot{R}(t,0) = (I-Q)^{-1}R$.

We can combine these results to conclude:

$$\hat{A}^* = \lim_{t\to\infty} \prod_{\tau=0}^{t} \hat{A}(\tau) = \lim_{t\to\infty} \tilde{A}(t,0) = \left[\begin{array}{c|c} \lim_{t\to\infty} \tilde{Q}(t,0) & \lim_{t\to\infty} \tilde{R}(t,0) \\ \hline 0 & I \end{array}\right] = \left[\begin{array}{c|c} 0 & (I-Q)^{-1}R \\ \hline 0 & I \end{array}\right] .$$

Given that $\hat{\boldsymbol{x}}^* = \hat{A}^* \hat{\boldsymbol{x}}(0)$ we can conclude Result (1) - that the signal mixes do converge. In particular:

$$\hat{\boldsymbol{x}}^* = \hat{A}^* \hat{\boldsymbol{x}}(0) = \left[\begin{array}{c|c|c} 0 & (I-Q)^{-1}R^+ & (I-Q)^{-1}R^- \\ \hline 0 & 1 & 0 \\ \hline 0 & 0 & 1 \end{array}\right] \left[\begin{array}{c} \hat{\boldsymbol{x}}(0) \\ \hline 1 \\ \hline 0 \end{array}\right] = \left[\begin{array}{c} (I-Q)^{-1}R^+ \\ \hline 1 \\ \hline 0 \end{array}\right] ,$$

that is, the steady state signal mixes of the agents not a function of the initial signals $\hat{x}(0)$. The signal mixes are instead entirely a function of the steady state orientations of the biased agents, encoded by the vector R^+, the edges from the positive biased agents to the positive ghost nodes.

Our remaining conclusions follow summarily from this. If all biased agents are negative ($\hat{y}^*_{\mathcal{B}} = 0$) R^+ is 0 and x^* is 0 for all agents. Inversely if all biased agents are positive ($\hat{y}^*_{\mathcal{B}} = 1$), x^* is 1 for all agents. For any other configuration of biased agents, the steady state is determined by the closed form $(I-Q)^{-1}R^+$. In this scenario $z^*_V > 0$ trivially as some biased nodes will be of the minority orientation. However, more crucially $z^*_R \geq 0$. That is, unbiased agents are no longer guaranteed to converge despite having no bias mechanism themselves. We investigate this and other properties of the unbiased agents in more detail in the next section.

4 Section S4: Steady state signal mix distribution

We now seek to approximate the distribution of signal mixes of the agents once the steady state is reached. We will first approximate the average steady state signal mix of each sub-population

in the network, followed by the steady state signal mix variance, and finally the full distribution itself. We will do this for the k-regular network case used in the body of the paper, and show via numerical simulations that it also captures the model's dynamics on more heterogeneous networks. Let us note that the results given in the following are the empirical distribution of the signal mixes for a given run of the model, as opposed to an ensemble over all possible runs of the model.

4.1 Steady state expected signal mix.

As detailed in 1, the model converges to a steady state $\hat{\mathbf{x}}^*$ which is entirely contingent on the settled orientation of the biased agents in the network. In what follows, we will calculate an approximation for the model's steady state expected signal mix *conditional* on a given fraction of positively oriented biased agents $f^+(t) = \hat{y}_{\mathcal{B}}(t)f$. We will then show how under some reasonable assumptions the "settled" value of $f^+(t)$ can be approximated from the initial orientation $f^+(0)$.

Consider an agent i picked uniformly at random at time t from the unbiased, positively oriented biased, negatively oriented biased sub-populations. Let us denote the signal mixes of agents belonging to such sub-populations as $\hat{x}_{i_{\mathcal{U}}}(t)$, $\hat{x}_{i_{\mathcal{B}+}}(t)$ and $\hat{x}_{i_{\mathcal{B}-}}(t)$, respectively. We are interested in establishing the expected steady state values for each of these quantities, denoted as

$$\hat{x}^*_{\mathcal{U}} = \lim_{t\to\infty} \mathbb{E}[\hat{x}_{i_{\mathcal{U}}}(t)] = \lim_{t\to\infty} \hat{x}_{\mathcal{U}}(t) \ ,$$

with analogous definitions for $\hat{x}^*_{\mathcal{B}+}$ and $\hat{x}^*_{\mathcal{B}-}$.

We begin by considering the sub-population of unbiased agents at some finite t. We note the following:

$$\hat{x}_{\mathcal{U}}(t+1) = \mathbb{E}[\hat{x}_{i_{\mathcal{U}}}(t+1)] = \mathbb{E}\left[\frac{\hat{x}_{i_{\mathcal{U}}}(t) + \sum_{j\in\partial_i}\hat{x}_j(t)}{k+1}\right] = \frac{\hat{x}_{\mathcal{U}}(t) + k\mathbb{E}[\hat{x}_j(t)]}{k+1} \ , \qquad (14)$$

where $\mathbb{E}[\hat{x}_j(t)]$ refers to the expected signal mix of a randomly picked agent j from the entire

population, which of course consists of the three aforementioned sub-populations. Therefore, we have

$$\begin{aligned}\mathbb{E}[\hat{x}_j(t)] &= (1-f)\mathbb{E}[\hat{x}_j(t)|j \in U] + f^+(t)\mathbb{E}[\hat{x}_j(t)|j \in \mathcal{B}^+(t)] + f^-(t)\mathbb{E}[\hat{x}_j(t)|j \in \mathcal{B}^-(t)] \qquad (15)\\ &= (1-f)\hat{x}_{\mathcal{U}}(t) + f^+(t)\hat{x}_{\mathcal{B}^+}(t) + f^-(t)\hat{x}_{\mathcal{B}^-}(t)\ .\end{aligned}$$

Plugging the above in (14) we get

$$\hat{x}_{\mathcal{U}}(t+1) = \frac{1}{k+1}\left[(1+k(1-f))\hat{x}_{\mathcal{U}}(t) + kf^+\hat{x}_{\mathcal{B}^+}(t) + kf^-\hat{x}_{\mathcal{B}^-}(t)\right]\ .$$

Repeating the above steps for positively oriented biased agents we get

$$\begin{aligned}\hat{x}_{\mathcal{B}^+}(t+1) &= \mathbb{E}[\hat{x}_{i_{\mathcal{B}^+}}(t+1)] = \mathbb{E}\left[\frac{\hat{x}_{i_{\mathcal{B}^+}}(t) + \sum_{j\in\partial_i}((1-w_i(t))\hat{x}_j(t) + w_i(t))}{k+1}\right]\\ &= \frac{\hat{x}_{\mathcal{B}^+}(t) + (1-q)k\mathbb{E}[\hat{x}_j(t)] + kq}{k+1}\ .\end{aligned}$$

where we have explicitly referenced the random variable $w_i(t)$ representing the fraction of successfully distorted negative signals (see 1), and made use of the fact that $\mathbb{E}[w_i(t)] = q$. We can use (15) again and write

$$\hat{x}_{\mathcal{B}^+}(t+1) = \frac{1}{k+1}\left[k(1-f)(1-q)\hat{x}_{\mathcal{U}}(t) + ((1-q)kf^+(t)+1)\hat{x}_{\mathcal{B}^+}(t) + (1-q)kf^-(t)\hat{x}_{\mathcal{B}^-}(t) + kq\right]\ ,$$

and similarly for negatively oriented biased agents:

$$\hat{x}_{\mathcal{B}^-}(t+1) = \frac{1}{k+1}\left[k(1-f)(1-q)\hat{x}_{\mathcal{U}}(t) + (1-q)kf^+(t)\hat{x}_{\mathcal{B}^+}(t) + ((1-q)kf^- + 1)(t)\hat{x}_{\mathcal{B}^-}(t)\right]\ .$$

We have therefore established the update rule for the expected signal mix of the three sub-populations at any time t. We collate this update rule into a matrix form for convenience:

$$\boldsymbol{\xi}(t+1) = \frac{1}{k+1}(\boldsymbol{F}(t) + \boldsymbol{I}_3)\boldsymbol{\xi}(t) + \boldsymbol{b}\,, \qquad (16)$$

where

$$\boldsymbol{\xi}(t) = [\hat{x}_{\mathcal{U}}(t), \hat{x}_{\mathcal{B}^+}(t), \hat{x}_{\mathcal{B}^-}(t)]^T\,, \qquad \boldsymbol{b} = \left[0, \frac{kq}{k+1}, 0\right]^T$$

and

$$\boldsymbol{F}(t) = k \begin{bmatrix} (1-f) & f^+(t) & f^-(t) \\ (1-q)(1-f) & (1-q)f^+(t) & (1-q)f^-(t) \\ (1-q)(1-f) & (1-q)f^+(t) & (1-q)f^-(t) \end{bmatrix} .$$

If we further simplify notation by defining $\hat{\boldsymbol{F}}(t) = (\boldsymbol{F}(t) + \boldsymbol{I}_3)/(k+1)$, we get to the following compact expression for (16):

$$\boldsymbol{\xi}(t+1) = \hat{\boldsymbol{F}}(t)\boldsymbol{\xi}(t+1) + \boldsymbol{b} .$$

The long-run evolution of the signal mixes can be determined from the above equation if the evolution of $f^+(t)$ (and, consequently, of $f^-(t)$) in the matrix $\hat{\boldsymbol{F}}(t)$ is known. Assume for the moment we are at some time t^* at which the system has settled, i.e., biased agents will keep their orientations intact and therefore will not cause the value of $f^+(t)$ to change for $t > t^*$. In this case we can write:

$$\lim_{t\to\infty} \boldsymbol{\xi}(t) = \lim_{t\to\infty} \hat{\boldsymbol{F}}(t^*)^t \boldsymbol{\xi}(t^*) + (\hat{\boldsymbol{F}}(t^*) - \boldsymbol{I}_3)^{-1}\boldsymbol{b} .$$

It can be shown easily that, due to its double substochasticity, we have $\lim_{t\to\infty} \hat{\boldsymbol{F}}(t^*)^t = 0$, and therefore

$$\lim_{t\to\infty} \boldsymbol{\xi}(t) = (\hat{\boldsymbol{F}}(t^*) - \boldsymbol{I}_3)^{-1}\boldsymbol{b} .$$

The above limit allows to calculate the steady state signal mixes for all sub-populations explicitly:

$$\lim_{t\to\infty} \boldsymbol{\xi}(t) = \begin{bmatrix} \hat{x}^*_{\mathcal{U}} \\ \hat{x}^*_{\mathcal{B}+} \\ \hat{x}^*_{\mathcal{B}-} \end{bmatrix} = \begin{bmatrix} f^+(t^*)/f \\ (1-q)f^+(t^*)/f + q \\ (1-q)f^+(t^*)/f \end{bmatrix} = \begin{bmatrix} \hat{y}_{\mathcal{B}}(t^*) \\ (1-q)\hat{y}_{\mathcal{B}}(t^*) + q \\ (1-q)\hat{y}_{\mathcal{B}}(t^*) \end{bmatrix} . \tag{17}$$

In the next Section we will approximate this result to the case where biased agents have not settled their orientation yet.

4.2 Predicting the trajectory of biased agents' orientations

Biased agents change their orientation when they receive a stream of incongruent signals that overcome their ability to distort them using confirmation bias. There are two points in the

evolution of the model where this is possible. Firstly, this may happen in the early stages of the evolution, where the information sets held by the agents are relatively small and the stochasticity of the model can induce changes in orientation. Secondly, this may happen in the long run, where sustained changes in orientation can be brought along when one of the two camps of biased agents becomes able to systematically bias the available information. This leads to the composition of signals experienced by each node to change consistently in one direction, which can cause large scale switches in orientation, which in turn triggers a domino effect, as newly switched nodes will accelerate the rate at which signals are distorted.

Let us capture this notion more formally. Consider the expected long term signal mix of each sub-population assuming the biased agents have settled ((17)). Suppose the positively oriented biased agents have an expected steady state signal mix $\hat{x}^*_{\mathcal{B}+} < 1/2$. If such steady state value is to be reached, then some positively oriented biased agents' signal mixes must fall below $1/2$, thereby switching orientation to negative. If this happens, then $\hat{y}_{\mathcal{B}}(t)$ falls and the steady state signal mix for *all* agents strictly decreases[5]. This, in turn, means more positively oriented biased agents switch orientation to reach their steady state, and so forth until all such agents switch to a negative orientation, yielding $\hat{y}_{\mathcal{B}} = 0$. A corresponding outcome can be determined for negatively oriented biased agents all being converted. We can therefore determine, for any given t^*, the approximate conditions under which we expect all positively oriented biased agents to switch their orientations to negative in the eventual steady state. Setting $\hat{x}^*_{\mathcal{B}+} < 1/2$ in (17) we have

$$(1-q)\hat{y}_{\mathcal{B}}(t^*) + q < \frac{1}{2} \qquad \Longrightarrow \qquad \hat{y}_{\mathcal{B}}(t^*) < \frac{1}{2-2q}\,, \tag{18}$$

and, correspondingly, for all negatively oriented biased agents to be tipped to positive we have

[5]This can be proven rigorously with the results from the previous Section: the steady state mix is $(I-Q)^{-1}R^+$. R^+ is a vector with 0 for each negative biased agent, and $(I-Q)^{-1}$ is element-wise > 0. A biased agent switching to positive turns a previous zero element of R^+ to positive, and adds another strictly positive vector to the steady state signal mix. The same argument is made in reverse for a positive to negative switch

the following condition:

$$\hat{y}_{\mathcal{B}}(t^*) > \frac{1-2q}{2-2q} \ . \tag{19}$$

Let us now consider the case $t_0 = 0$, which means we are approximating the expected trajectory of the entire system given a starting fraction of positively oriented biased agents $f^{+*}(0)/f = \hat{y}_{\mathcal{B}}(0)$. We then have the following approximate result for the steady state signal mix of the *unbiased* sub-population:

$$\hat{x}^*_{\mathcal{U}} = \hat{y}^*_{\mathcal{B}} \approx \begin{cases} \hat{y}_{\mathcal{B}}(0) & \text{for } \frac{1}{2(1-q)} \leq \hat{y}_{\mathcal{B}}(0) \leq \frac{1-2q}{2(1-q)} \\ 1 & \text{for } \hat{y}_{\mathcal{B}}(0) > \frac{1-2q}{2(1-q)} \\ 0 & \text{for } \hat{y}_{\mathcal{B}}(0) < \frac{1}{2(1-q)} \ , \end{cases}$$

where the latter two conditions derive from Eqs. (18) and (19), while the first condition is the same reported in (17) adapted for the case $t_0 = 0$.

4.3 Steady state signal mix variance.

In the previous Section we have provided approximations for the first moment of the steady state signal mixes of the unbiased agents, as well as those of the two biased agent sub-populations. We have also approximated the long term "settled" fractions of positively and negatively oriented biased agents. We noted that for $\hat{y}_{\mathcal{B}}(0) > \frac{1-2q}{2(1-q)}$ ($\hat{y}_{\mathcal{B}}(0) < \frac{1}{2(1-q)}$), the steady state signal mix is likely to asymptotically reach 1 (0). Under these conditions, all agents eventually trivially possess the same signal $+1$ (-1). Therefore the distribution of signal mixes tends asymptotically to a Dirac distribution on 1 (0).

We therefore proceed with the assumption that $\frac{1}{2(1-q)} \leq \hat{y}_{\mathcal{B}}(0) \leq \frac{1-2q}{2(1-q)}$, and as such use (17) to approximate:

$$\lim_{t\to\infty} \boldsymbol{\xi}(t) = \begin{bmatrix} \hat{y}_{\mathcal{B}}(t^*) \\ (1-q)\hat{y}_{\mathcal{B}}(t^*) + q \\ (1-q)\hat{y}_{\mathcal{B}}(t^*) \end{bmatrix} \approx \begin{bmatrix} \hat{y}_{\mathcal{B}}(0) \\ (1-q)\hat{y}_{\mathcal{B}}(0) + q \\ (1-q)\hat{y}_{\mathcal{B}}(0) \end{bmatrix}$$

Correspondingly, we note $f^{+*} = \hat{y}_{\mathcal{B}}(t^*) = \hat{y}_{\mathcal{B}}(0)$. We would now like to characterise the distribution of signal mixes for each sub-population at the steady state beyond its first moment.

We begin with an approximation of the variance, under the asymptotic limit of large populations $n \to \infty$.

For convenience we define the steady state signal mix variance of any sub-population G as σ_G^2. In the following, we will provide approximate expressions for the steady state signal mix variances $\sigma_{\mathcal{U}}^2$, $\sigma_{\mathcal{B}^+}^2$, and $\sigma_{\mathcal{B}^-}^2$, and for the overall variance σ_V^2.

Consider an agent i picked uniformly at random from the entire population. The variance of such an agent's steady state signal mix $\mathrm{Var}[\hat{x}_i^*]$ represents the variance across the entire population σ_V^2. From the law of total variance, this can be broken down as follows

$$\sigma_V^2 = \mathrm{Var}[\hat{x}_i^*] = \mathbb{E}\big[\mathrm{Var}[\hat{x}_i^*] \mid i \in \{\mathcal{U}, \mathcal{B}^+, \mathcal{B}^-\}\big] + \mathrm{Var}\big[\mathbb{E}\left[\hat{x}_i^* \mid i \in \{U, \mathcal{B}^+, \mathcal{B}^-\}\right]\big] , \tag{20}$$

where

$$\mathbb{E}\big[\mathrm{Var}[\hat{x}_i^*] \mid i \in \{\mathcal{U}, \mathcal{B}^+, \mathcal{B}^-\}\big] = (1-f)\sigma_{\mathcal{U}}^2 + f^{+*}\sigma_{\mathcal{B}^+}^2 + f^{-*}\sigma_{\mathcal{B}^-}^2 , \tag{21}$$

and

$$\begin{aligned}
\mathrm{Var}\big[\mathbb{E}\left[\hat{x}_i^* \mid i \in \{\mathcal{U}, \mathcal{B}^+, \mathcal{B}^-\}\right]\big] &= (1-f)\left\{\frac{f^{+*}}{f} - \mathbb{E}\Big[\mathbb{E}[\hat{x}_i^* \mid i \in \{\mathcal{U}, \mathcal{B}^+, \mathcal{B}^-\}]\Big]\right\}^2 \\
&+ f^{+*}\left\{(1-q)\frac{f^{+*}}{f} + q - \mathbb{E}\Big[\mathbb{E}[\hat{x}_i^* \mid i \in \{\mathcal{U}, \mathcal{B}^+, \mathcal{B}^-\}]\Big]\right\}^2 \\
&+ f^{-*}\left\{(1-q)\frac{f^{+*}}{f} - \mathbb{E}\Big[\mathbb{E}[\hat{x}_i^* \mid i \in \{\mathcal{U}, \mathcal{B}^+, \mathcal{B}^-\}]\Big]\right\}^2 .
\end{aligned} \tag{22}$$

Noting that

$$\mathbb{E}\Big[\mathbb{E}[\hat{x}_i^* \mid i \in \{\mathcal{U}, \mathcal{B}^+, \mathcal{B}^-\}]\Big] = (1-f)\frac{f^{+*}}{f} + f^{+*}\left((1-q)\frac{f^{+*}}{f} + q\right) + f^{-*}\left((1-q)\frac{f^{+*}}{f}\right) = \frac{f^{+*}}{f}$$

we can considerably simplify (22):

$$\mathrm{Var}\big[\mathbb{E}\left[\hat{x}_i^* \mid i \in \{\mathcal{U}, \mathcal{B}^+, \mathcal{B}^-\}\right]\big] = \frac{q^2 f^{+*} f^{-*}}{f} = f q^2 \hat{x}_{\mathcal{U}}^*(1-\hat{x}_{\mathcal{U}}^*) ,$$

where in the last step we have used the fact that $\hat{x}_{\mathcal{U}}^* = f^{+*}/f$, as per (17).

(22) provides a compact expression for the second contribution for the overall variance σ_V^2 in (20). We now turn to the first term ((21)). In order to be able to calculate it, we must compute the variance of each sub-population. Let us begin with the unbiased agent sub-population:

$$\sigma_{\mathcal{U}}^2 = \mathrm{Var}[\hat{x}_{\mathcal{U}}^*] = \mathrm{Var}\left[\frac{1}{k}\sum_{j\in\partial_i}\hat{x}_j^*\right] = \frac{\sigma_V^2}{k} + \frac{2}{k^2}\sum_{(j,\ell)\in\partial_i}\mathrm{Cov}[\hat{x}_j^*, \hat{x}_\ell^*] = \frac{\sigma_V^2}{k} + \mathcal{O}\left(\frac{1}{k^2}\right) , \quad (23)$$

where in the last term we have assumed the covariance term to decay as k^{-2}, which will be proved in the next Section. In analogy with the above results, for the biased agent sub-population (either positively or negatively oriented) we have:

$$\sigma_{\mathcal{B}}^2 = \mathrm{Var}[\hat{x}_{i_B}^*] = \frac{(1-q)^2\sigma_V^2}{k} + \mathcal{O}\left(\frac{1}{k^2}\right) . \quad (24)$$

Substituting the two expressions above in (21), and combining the result with the one obtained in (22), we finally obtain the following result for the overall variance in (20):

$$\sigma_V^2 = \frac{1 - fq(2-q)}{k}\sigma_V^2 + fq^2\hat{x}_{\mathcal{U}}^*(1-\hat{x}_{\mathcal{U}}^*) + \mathcal{O}\left(\frac{1}{k^2}\right) .$$

Solving for σ_V^2 we get

$$\sigma_V^2 = \frac{kfq^2\hat{x}_{\mathcal{U}}^*(1-\hat{x}_{\mathcal{U}}^*)}{k + fq(2-q) - 1} + \frac{k}{k + fq(2-q) - 1}\mathcal{O}\left(\frac{1}{k^2}\right) \approx fq^2\hat{x}_{\mathcal{U}}^*(1-\hat{x}_{\mathcal{U}}^*) ,$$

where we have used the fact that $fq(2-q) - 1 \in [-1, 0]$, and therefore $fq(2-q) - 1 \ll k$ even for moderate connectivity.

Finally, we can specialize the above result to the three sub-populations via Eqs. (23) and (24):

$$\sigma_{\mathcal{U}}^2 \approx \frac{fq^2\hat{x}_{\mathcal{U}}^*(1-\hat{x}_{\mathcal{U}}^*)}{k} \quad (25)$$

$$\sigma_{\mathcal{B}_\pm}^2 \approx \frac{f\left(q(1-q)\right)^2\hat{x}_{\mathcal{U}}^*(1-\hat{x}_{\mathcal{U}}^*)}{k} . \quad (26)$$

4.4 Explicit neighbourhood covariance expressions.

In this Section we establish that the covariance term appearing in (23) can indeed be assumed to be of order k^{-2}. The first thing to note is that the term $\mathrm{Cov}[\hat{x}_j^*, \hat{x}_\ell^*]$ for two generic agents

can be bounded above by the covariance $\mathrm{Cov}[\hat{x}_j^*, \hat{x}_\ell^* | j, \ell \in U]$ between unbiased agents' steady state signal mixes. To see this, suppose $j, \ell \in \mathcal{B}^+$:

$$\begin{aligned}\mathrm{Cov}[\hat{x}_j^*, \hat{x}_\ell^* | j, \ell \in \mathcal{B}^+] &= \mathrm{Cov}\left[\frac{(1-q)\sum_{h\in\partial_j}\hat{x}_h^* + qk}{k}, \frac{(1-q)\sum_{m\in\partial_\ell}\hat{x}_m^* + qk}{k}\right] \\ &= \frac{(1-q)^2}{k^2}\mathrm{Cov}\left[\sum_{h\in\partial_j}\hat{x}_h^*, \sum_{m\in\partial_\ell}\hat{x}_m^*\right] < \frac{1}{k^2}\mathrm{Cov}\left[\sum_{h\in\partial_j}\hat{x}_h^*, \sum_{m\in\partial_\ell}\hat{x}_m^*\right] \\ &= \mathrm{Cov}[\hat{x}_j^*, \hat{x}_\ell^* | j, \ell \in U] \,.\end{aligned}$$

Let $\mathrm{Cov}(\hat{x}_j^*, \hat{x}_l^* | (j, l) \in U) = \sigma_2$, denote the least upper bound for the covariance between two

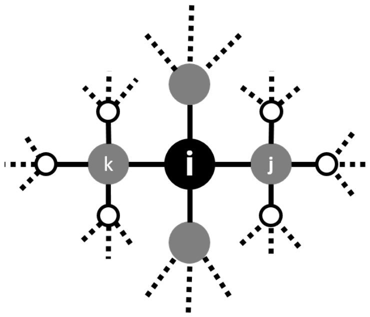

Figure S4: The covariance of i's neighbours, j and k (grey), can be decomposed into the covariance between its neighbours (black and white). This consists of the covariance between neighbours that are two steps apart (black to white) as well as those that have distance four steps apart (white to white).

nodes of distance 2 apart. Similarly, let σ_d be the same for nodes of distance d apart. In the remainder of this section we are seeking to establish a relationship between these *upper bounds* and in doing so recursively determine the upper bound at $d = 2$.

It is worth reiterating that we are approximating the variance of the steady state signal mixes at the asymptotic limit $n \to \infty$. Given this assumption, a k-regular tree will approximate a Cayley tree. A useful consequence of this assumption is that the network is globally tree-like, and loops vanish in the limit. As such, only a single path exists between any two nodes. Therefore, in (23) we have:

$$\sigma_{\mathcal{U}}^2 = \frac{\sigma_V^2}{k} + \frac{2}{k^2}\sum_{(j,\ell)\in\partial_i}\mathrm{Cov}[\hat{x}_j^*, \hat{x}_\ell^*] \leq \frac{\sigma_V^2}{k} + \frac{2}{k^2}\sum_{(j,\ell)\in\partial_i}\sigma_2 = \frac{\sigma_V^2}{k} + \frac{\sigma_2}{k}(k-1) \,.$$

Therefore we can establish that if $\sigma_2 = \mathcal{O}(k^{-2})$, the whole expression will be of order $\mathcal{O}(k^{-2})$. To do this note:

$$\begin{aligned}
\text{Cov}[\hat{x}_j^*, \hat{x}_\ell^*] &= \frac{1}{k^2}\text{Cov}\left[\hat{x}_i^* + \sum_{m\in\partial_j/i} \hat{x}_m^*, \hat{x}_i^* + \sum_{n\in\partial_\ell/i} \hat{x}_n^*\right] = \frac{1}{k^2}\left(\text{Cov}[\hat{x}_i^*, \hat{x}_i^*] + \sum_{(m,n)\in[\partial_j\times\partial_\ell]/(i,i)} \text{Cov}[\hat{x}_m^*, \hat{x}_n^*]\right) \\
&= \frac{1}{k^2}\left(\text{Cov}[\hat{x}_i^*, \hat{x}_i^*] + \sum_{m\in\partial_j/i}\text{Cov}[\hat{x}_i^*, \hat{x}_m^*] + \sum_{n\in\partial_\ell/i}\text{Cov}[\hat{x}_i^*, \hat{x}_n^*] + \sum_{(m,n)\in[\partial_j/i\times\partial_\ell/i]}\text{Cov}[\hat{x}_m^*, \hat{x}_n^*]\right) .
\end{aligned}$$

In this last step, we explicitly break down the covariance sum into the covariance between the neighbours of j and l, which exist at various distances to one another. This is illustrated in S4.

We can group these covariance pairs by their distance and bound them using our defined bounds σ_d:

$$\text{Cov}[\hat{x}_j^*, \hat{x}_\ell^*] \leq \frac{1}{k^2}\left(\sigma_{\mathcal{U}}^2 + 2(k-1)\sigma_2 + (k-1)^2\sigma_4\right) .$$

This allows us to recursively define

$$\sigma_2 = \frac{1}{k^2}\left(\sigma_{\mathcal{U}}^2 + 2(k-1)\sigma_2 + (k-1)^2\sigma_4\right) .$$

Re-arranging this expression we get:

$$\sigma_2 = \frac{1}{(k-1)^2+1}\sigma_{\mathcal{U}}^2 + \frac{(k-1)^2}{(k-1)^2-1}\sigma_4 = \frac{1}{(k-1)^2+1}\sigma_0 + \frac{(k-1)^2}{(k-1)^2-1}\sigma_4 .$$

In the final step, we have replaced $\sigma_{\mathcal{U}}^2$ with σ_0 - which emphasizes that this term is merely the covariance of a node with a node at distance 0 (i.e. its own variance), and $\sigma_{\mathcal{U}}^2$ is the largest possible variance expression amongst the biased and unbiased nodes. We can easily (though quite tediously) repeat this process for the covariance of nodes at any distance d to establish:

$$\sigma_d = \frac{1}{(k-1)^2+1}\sigma_{d-2} + \frac{(k-1)^2}{(k-1)^2-1}\sigma_{d+2} .$$

This linear recurrence relation can be solved with the boundary conditions that $\sigma_0 = \sigma_{\mathcal{U}}^2$ and $\lim_{d\to\infty}\sigma_d = 0$[6]. This establishes:

$$\sigma_d = \frac{\sigma_{\mathcal{U}}^2}{(k-1)^d} ,$$

[6]In other words, nodes at infinitely long distances have a covariance that decays to zero

and therefore:

$$\sigma_2 = \frac{\sigma_{\mathcal{U}}^2}{(k-1)^2} = \mathcal{O}(k^{-2}) \; .$$

Therefore, to finalise (23):

$$\sigma_{\mathcal{U}}^2 = \frac{1}{k}\sigma_V^2 + \mathcal{O}\left(\frac{1}{k^2}(k^2 - k)\sigma_2\right) = \frac{1}{k}\sigma_V^2 + \mathcal{O}(k^{-2}) \; .$$

4.5 Steady state signal mix normality.

We finally proceed to demonstrate that the distribution of signal mixes is approximately normal when k and n are large. Assume firstly that $n \to \infty$, which ensures that the model's k-regular network becomes a Cayley tree with no loops. Let us also assume that $k = \epsilon n$ for some $\epsilon > 0$, ensuring k also grows arbitrarily large, but still can be arbitrarily smaller than n.

As we have already established in the previous section, the covariance between the steady state signal mixes of unbiased agents at distance 2 decays as k^{-2}, which implies that such signals mixes become asymptotically independent in the aforementioned limits. Therefore, the steady state signal mix of an unbiased agent $\hat{x}_{i\mathcal{U}}^* = \sum_{j\in\partial_i} \hat{x}_j^*/k$ becomes the sum of an infinitely large set of independent random variables. Furthermore, the variables will follow one of three distributions, depending on which sub-population the agent's neighbors belong to:

$$\hat{x}_{i\mathcal{U}}^* = \frac{1}{k}\sum_{j\in\partial_i} \hat{x}_j^* = \frac{1}{k}\left(\sum_{j\in\partial_i\cap U} \hat{x}_j^* + \sum_{j\in\partial_i\cap\mathcal{B}^+} \hat{x}_j^* + \sum_{j\in\partial_i\cap\mathcal{B}^-} \hat{x}_j^*\right) \; .$$

Each of the above contributions is a sum of an infinitely large set of independent and identically distributed variables, which implies that each of them is normally distributed. This, in turn, implies that the steady state signal mixes of the unbiased agents (and, by generalisation, of the biased agents) is asymptotically normal. From the results obtained for the first two moments of the signal mix distributions in the previous sections (see Eqs. (17) and (25)), we can conclude

that when $n \to \infty$ and $k = \epsilon n$ we have

$$
\begin{aligned}
\hat{x}_{\mathcal{U}} &\xrightarrow{d} \mathcal{N}\left(\hat{x}^*_{\mathcal{U}}, \frac{fq^2\hat{x}^*_{\mathcal{U}}(1-\hat{x}^*_{\mathcal{U}})}{k}\right) \\
\hat{x}_{\mathcal{B}^+} &\xrightarrow{d} \mathcal{N}\left((1-q)\hat{x}^*_{\mathcal{U}} + q, \frac{f\left(q(1-q)\right)^2\hat{x}^*_{\mathcal{U}}(1-\hat{x}^*_{\mathcal{U}})}{k}\right) \\
\hat{x}_{\mathcal{B}^-} &\xrightarrow{d} \mathcal{N}\left((1-q)\hat{x}^*_{\mathcal{U}}, \frac{f\left(q(1-q)\right)^2\hat{x}^*_{\mathcal{U}}(1-\hat{x}^*_{\mathcal{U}})}{k}\right) .
\end{aligned}
$$

The normality of the distribution for the unbiased agents is demonstrated in Figure 3 of the main text.

5 Section S5: Accuracy

We can now aggregate our results in order to approximate the accuracy of a social network. As described in the main body of the paper, the accuracy of a network $\mathcal{A}(G)$ is the expected fraction of accurate unbiased agents in the steady state, i.e. accuracy quantifies the probability $\mathrm{Prob}(y_{i_{\mathcal{U}}} = +1)$ that a randomly picked unbiased agent in a random realisation of the model will correctly learn the ground truth[7].

This is of course a complex outcome determined by a dynamic series of processes worth recapping. First, the model will generate initial signals for all agents, both biased and unbiased. All agents will share their signals, but biased agents will selectively sample incoming signals based on their current orientation. Over time, biased agents are able to influence the set of signals in the system, and the system converges towards a steady state where each agent possesses an equilibrium mix of signals. Accurate agents are those whose equilibrium signal mix is contains more positive than negative signals, i.e. $x^*_i > 1/2$.

In the previous Sections we have calculated the distribution of the initial signals, as well as the approximate steady state signal mix for a given set of initial signals (see Equation 4.2). We have also approximated the steady state individual signal mix distributions (see Eq. 27), and

[7]The definition of accuracy could very easily be extended to all agents instead of just unbiased agents, but we retain discussion to unbiased agents for simplicity

as such we can approximate the fraction of accurate unbiased agents for a given steady state, which reads

$$A(\mathcal{G}) = \frac{1}{2}\int_0^1 dx^*_{\mathcal{U}}\; P(x^*_{\mathcal{U}})\,\mathrm{erfc}\left(\frac{\frac{1}{2} - x^*_{\mathcal{U}}}{\sqrt{2}\,\sigma_{\mathcal{U}}}\right) \,, \tag{27}$$

where $P(x^*_{\mathcal{U}})$ is the distribution of the average signal mix across unbiased agents as determined by (4.2) (see Eq. (3) of the main paper), while the complementary error function quantifies the fraction of unbiased agents whose steady state signal mix is above $1/2$, and are therefore accurate, under the normal approximation outlined in the previous section.

Both ingredients employed in (27) have been obtained based on a number of approximations and asymptotic assumptions. We checked how such approximations hold against numerical simulations of the model's dynamics. The results are shown in Fig. S5 both for k-regular and Erdős-Rényi networks. As can be seen, the average accuracy obtained across independent numerical simulations of the model closely matches the expected value obtained with (27), even for relatively low network size and average degree (the results reported were obtained for $n = 10^4$ and $k = 8$). The wider error bars for lower f reflect the expected outcome that most runs of the model will result in total consensus on either $X = \pm 1$ (and therefore all accurate or all inaccurate agents), whereas as f grows, the agents are highly polarised and the fraction of accurate and inaccurate agents will be relatively constant.

6 Section S6: Regression results

6.1 Theory and model interpretation.

Our model is stylized, and therefore largely agnostic as to a particular interpretation of its parameters. Nevertheless, it is quite well suited to provide an initial exploration on a number of issue. In this Section, we shall test the model's ability to shed light on the impact that Internet access has on shaping popular opinion on specific issues (global warming in this case). In order to do this, we first specify how we are going to relate our model's parameters to real-world measurable quantities.

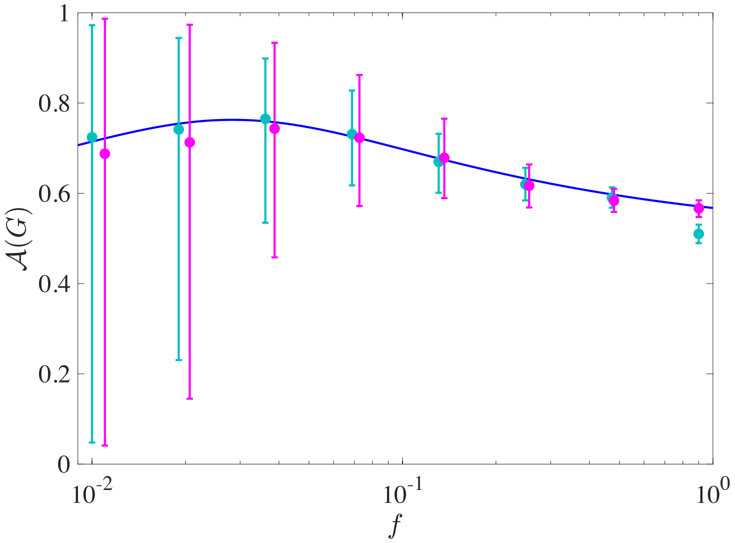

Figure S5: Non-monotonic changes in expected accuracy as f increases. The model's prediction are compared to numerical results obtained with simulations on both k-regular (light blue) and Erdős-Rényi (purple) networks. The parameters used in the simulations were $n = 10^4$, $p = 0.53$, $k = 8$, $q = 1$.

There are two convenient (and pragmatically equivalent) interpretations of the model in the context of Internet use. Consider the agent-specific ghost node interpretation, where each ghost node attached to a biased agent represents an aggregation of the "filter bubble" (passive algorithmic affects) and "selective exposure" (actively selecting information in a biased way) effects. An increase in Internet access therefore translates to an increase in access to these self-confirmatory effects, and corresponds to changing unbiased agents into biased agents (an increase in f). Alternatively, one could consider a scenario where the fraction of biased agents is fixed, in which case an increase in Internet would improve their ability to obtain self-confirmatory information (an increase in q). For the purposes of this exploration, however, the two effects are equivalent, and for convenience we only retain the interpretation where f increases.

As far as the interpretation of the degree variable k is concerned, the important distinction to make here is that we are not interested in "social networks" as a catch-all term for the number of family and friends one has. Rather, given the model, we are interested in the degree to which

individuals actively exchange information with their underlying social network with regards to the topic of interest. Therefore, for k we wish to measure the volume of active social information diffusion in a given population.

As per (25), one of our model's main results is that f and k work in opposite directions when it comes to polarisation[8] - an increase in confirmatory behaviours increases polarisation and is equivalent to a reduction in social information. Furthermore, if the majority of the population accurately learns the ground truth ($x^*_{\mathcal{U}} > 1/2$), reductions in polarisation can be translated to an increase in consensus on the truth, as a smaller fraction of the population will arrive at inaccurate beliefs.

Translated to current research on the role of the Internet, we attempt to use our model to shed light on what has been thought of as the dichotomous effects of Internet access on social learning and polarisation. On one hand it has been argued that Internet access improves exposure to diverse information via social networks [2, 3, 4], whereas on another it has been argued that Internet access enables confirmation bias on a previously unprecedented scale [5, 6]. These contradictory effects may be in part responsible for the range of conflicting results obtained in recent research, and in our closing remarks we revisit some of these existing results in the context of our model.

6.2 Data sources and measuring variables

In order to test the model's predictions in the aforementioned context, we gathered data from the Yale Programme on Climate Change Communication 2016 Opinion Maps [7], which provides state and county level survey data on opinions on global warming, as well as behaviours such as the propensity to discuss climate change with friends and family. We combined this with FCC 2016 county level data on residential high speed Internet access [8]. Finally, we also used a supplemental source in the data aggregated by the Joint Economic Council's Social

[8]Strictly speaking the result refers to the variance in information sets, but we exploit the monotonic relationship between information variance $\sigma^2_{x^*_{\mathcal{U}}}$ and polarization $z^*_{\mathcal{U}}$ for the remainder of this section

Capital Project [9], a government initiative aiming to measure social capital at a county level by aggregating a combination of state and county level data from sources such as the American Community Survey, the Current Population Survey, and the IRS.

In this context, we measured accuracy as the estimated fraction of the population believing that “global warming is happening”. We refer to this as “GW Accuracy”. In other words, we are attempting to examine the degree to which social information and access to confirmatory bias mechanisms affect the ability of individuals to accurately learn an objective, measurable and uncontroversial ground truth (that global temperatures are rising).

Internet access is measured by the FCC’s data on county-level high speed broadband penetration amongst residentials (in [5], the authors utilise another instrumental variable approach to argue that increased broadband penetration does in fact increase Internet use). In Table S1 we demonstrate preliminary ordinary least squares regression results by regressing GW Accuracy on Internet access, accounting for a range of covariates such as median age, median income, county population size and the fraction of adults with college degrees. The results indicate that even after controlling for relevant covariates, the net effect of Internet access on accuracy is positive[9] (and by interpretation, the effect of polarisation on this particular ground truth is negative).

However, this alone is insufficient as research indicates Internet access is likely to improve the degree to which individuals can communicate information to friends and family, which in our model is precisely the variable k. The Yale Climate Change data includes a measure estimating the fraction of the county population that discusses global warming regularly with family

[9]One may note that the impact of median income on this regression, and all subsequent results. is negative. We have verified this result through a number of additional checks. It appears that the inclusion of college education heavily affects this coefficient, implying that the effect of income on global warming beliefs is heavily mediated by access to education. We also performed some further checks by including dummy variables for political partisanship using county level voting results for the 2016 presidential elections. While political partisanship provides additional explanatory power over and above the current set of variables, the coefficient for income when including it is still negative. Unpacking the exact nature of this relationship would require a broader range of economic and political factors, which is clearly outside the scope this initial analysis, so we exclude partisanship and continue with the original model, allowing the coefficients to be taken at face value.

and friends ("Social Discussion"). To sense check this, Table S1 (column 2) demonstrates that increased Internet access does indeed improve the ability to discuss matters with friends and family, even after controlling for relevant covariates, which is consistent with a broad set of empirical research on the topic (see [10] for a review).

Therefore, this allows us to construct our final model in Table 1(3) where we regress GW Accuracy on *both* Social Discussion and Internet access (and the covariates). We can now interpret the coefficient on Internet access as the residual effect of Internet access *after* controlling for the effect it has on Social Discussion. One way of thinking about this is to consider all causal pathways from Internet access to belief formation - some fraction of them will be via improved access to social and discussion networks (communication platforms, online social networks, and forums), and the remaining fraction will be non-social (algorithmic effects, filter bubbles, online news media, selective exposure, etc). By accounting for the former effects by observing the discussion network size in Social Discussion, the residual effect of internet access will aggregate all these other effects. This lines up with the interpretation of f in our model - Internet users will have access to these effects ("biased agents") and non-Internet users will not. The results confirm our hypothesis - Social Discussion (k) and residual Internet Access (f) act in opposite directions when it comes to learning the ground truth, even after conditioning on a range of covariates.

It is worth unpacking these results in detail. The direct effect of a 1 percentage point increase in Internet access on global warming accuracy is negative[10] (-2.400). The direct effect on social discussion is extremely positive[11] (3.736), which leads to a corresponding improvement in accuracy[12] of $1.057 \times 3.736 \approx 3.95$. The net effect, of course, is positive ($3.95 - 2.40 = 1.55$), as indicated in the original, simple regression[13]. However, breaking down the causal mechanism into its constituent elements - direct internet use effects vs socially mediated internet effects -

[10]Table 1, Column 3, Row 2.
[11]Table 1, Column 2, Row 2.
[12]Table 1, Column 3, Row 1.
[13]Table 1, Column 1, Row 2.

Table S1: Initial Regression Results

	Dependent variable:		
	GW Accuracy	Social Discussion	GW Accuracy
	(1)	(2)	(3)
Social Discussion			1.057^{***}
			(0.019)
Internet Access	1.550^{**}	3.736^{***}	-2.400^{***}
	(0.664)	(0.447)	(0.471)
Median Age	-0.044^{***}	-0.020^{*}	-0.023^{*}
	(0.017)	(0.011)	(0.012)
log(Median Household Income)	-6.691^{***}	-1.659^{***}	-4.938^{***}
	(0.464)	(0.313)	(0.327)
log(Total Pop)	0.690^{***}	-0.399^{***}	1.112^{***}
	(0.071)	(0.048)	(0.050)
College Education	0.335^{***}	0.304^{***}	0.013
	(0.013)	(0.009)	(0.011)
Constant	123.417^{***}	43.730^{***}	77.178^{***}
	(4.846)	(3.267)	(3.501)
Observations	2,933	2,933	2,933
R^2	0.312	0.448	0.662
Adjusted R^2	0.311	0.447	0.661
Residual Std. Error	4.395 (df = 2927)	2.963 (df = 2927)	3.082 (df = 2926)
F Statistic	265.316^{***}	474.324^{***}	953.601^{***}
	(df = 5; 2927)	(df = 5; 2927)	(df = 6; 2926)
Note:	$^{*}p$ <0.1; $^{**}p$ <0.05; $^{***}p$ <0.01		

allows us the capture the nuance of what is actually happening.

6.3 Accounting for simultaneous causality.

A clear shortcoming of the above analysis is the fact that the variable "Social Discussion" is likely to have a reverse causal relationship with the outcome variable of "GW Accuracy". That is, the more likely individuals are to believe global warming is happening, the more likely they are to discuss this topic with friends and family.

In order to account for this, we will take an instrumental variable approach. That is, we need some instrument that can account for independent variation in discussion with family and friends, which is otherwise unlikely to affect the belief in global warming. We note as before that k can be interpreted as the fraction of the "underlying social network" that is activated to transmit social information related to the topic of global warming. We are therefore interested in a variable that can measure the pre-existing strength of these underlying social networks. To do so, we make use of the Social Capital Project, a government research programme by the Joint Economic Committee that attempts to measure Social Capital at a state and county level throughout the US. Social Capital as defined in this study (and numerous others[14]) refers broadly to something "related to social relationships, social networks, and civil society". More specifically, it is measured with an intention to reflect communities with "an abundance of close, supportive relationships" [9].

The index itself measures a spectrum of factors, and in particular a "Community Health" subindex. The subindex is calculated as the leading principal component across a variety of state and county-level measures of community engagement (where people ostensibly meet and socialise with friends and family), including religious congregations, non-religious non-profit activities, public meeting attendance, working with neighbours to fix things, attending a meeting where politics was discussed, etc. This index is then validated by examining bivariate correlations with a battery of county level benchmarks and measures of social dysfunction.

The strength of this instrument is established in Table S2 (column 1), where a first stage least squares regression is run to show that improvements in Community Health do translate to improved discussion with friends and family (controlling for covariates).

The validity is established through a series of additional checks. Factors such as religious attendance, public meetings, etc. are unlikely to have a causal effect on people's beliefs about global warming independent of them being a medium to allow for social discussion of these

[14] i.e. Putnam [11] (1995, p.19), "...social capital refers to connections among individuals' social networks and the norms of reciprocity and trustworthiness that arise from them".

topics. The only other reasonable and plausibly significant causal channel is if these factors are caused by or cause an increase membership in social groups (for instance, political parties) that are strongly associated with reduced belief in global warming. In particular, it is well-established that members of the Republican Party have a reduced belief in the existence of Global Warming [12]. To check this, we examined the bivariate correlation between Community Health and the percentage of GOP votes cast in the 2016 presidential election. The results were weak, with a correlation of only 0.14, meaning only 1.8% of the variation in the measures were explained by the relationship.

Having established the strength and validity of the instrument, we demonstrate the results from the two stage least squares regression results in Table S2 (column 2). We can see the qualitative results of the simpler model have been preserved, with the effects predictably attenuated. However, the results are still significant, and corroborate our theory. After separating out the social and confirmatory effects of Internet access, we can see the impact on Accuracy (and Polarisation) both occur in the direction that we predict.

Once again, let us unpack the results. The direct effect of a 1 percentage point increase in internet access on global warming accuracy is negative[15] (-1.712). The direct effect on social discussion is extremely positive[16] (3.143), which leads to a corresponding improvement in accuracy[17] of $0.872 \times 3.143 \approx 2.74$. The net effect, of course, is positive $(2.74 - 1.71 = 1.03)$. Once again, breaking down the causal mechanism into its constituent elements - direct internet use effects vs socially mediated internet effects - allows us the capture the nuance of what is actually happening.

It appears, for the topic of global warming, the net impact of Internet access on social learning is positive. Increase in Internet access has a direct negative impact on learning (via f, or q). However, it leads to a significant positive impact on social discussion (k), and the

[15]Table 2, Column 2, Row 3.
[16]Table 2, Column 1, Row 2.
[17]Table 2, Column 2, Row 2.

net result of this is positive. This result remains robust even after controlling for a battery of relevant covariates.

It should be emphasized that this result is merely an initial exploration of how our model can provide some testable predictions to empirical data, as opposed to a detailed effort to understand the effect of Internet access on global warming beliefs. Having said that, the initial results are encouraging, and we hope the clarity of the analytic results of our model pave the way for testing variations of the idea of biased information aggregation in a range of outcomes and settings.

6.4 Making sense of broader empirical results.

We have seen so far that our model can help us decompose the effect of internet access on learning in the specific case of global warming facts. We now see if the model can help us better understand the seemingly conflicting findings we have found in existing research as indicated above. It should be said that the following interpretations are meant only to be indicative of how our model can help shape our theoretical understanding of empirical phenomena, rather than a detailed exploration of the specific empirical questions these papers explore.

In [13], the authors argue that internet access has not had an effect on political polarisation because the demographic with the lowest increase in internet use - the elderly - has had the highest increase in political polarisation. However, it is also well established that older people have smaller network sizes than younger people [14] and growing evidence of demographic shifts suggest that older people are increasingly living alone [15]. This translates to a direct fall in k for such populations, and without a corresponding increase in k provided by internet access, we would in fact expect to see higher polarisation in such a group.

In [5], the authors argue that an increase in internet access leads to an increase in political polarisation. Firstly, it is worth noting that the overall effect size is very small - increasing the number of broadband providers by 10% increases political polarisation by 0.003 points (on a scale between 0 and 1). This is consistent with notion that social connectivity will dampen the

Table S2: IV Regression Results

	Dependent variable:	
	Social Discussion	GW Accuracy
	OLS *(First Stage LS)*	*instrumental* *variable (2SLS)*
	(1)	(2)
Community Health Index	1.501***	
	(0.081)	
Social Discussion		0.872***
		(0.060)
Internet Access	3.143***	-1.712***
	(0.424)	(0.523)
log(Median Household Income)	-1.814***	-5.281***
	(0.297)	(0.346)
log(Total Pop)	0.300***	1.044***
	(0.059)	(0.056)
Median Age	-0.081***	-0.028**
	(0.011)	(0.012)
College Education	0.245***	0.070***
	(0.009)	(0.021)
Constant	42.621***	85.649***
	(3.095)	(4.356)
Observations	2,932	2,932
R^2	0.506	0.651
Adjusted R^2	0.505	0.651
Residual Std. Error (df = 2925)	2.803	3.129
F Statistic	499.387*** (df = 6; 2925)	
Note:		$^{*}p$ <0.1; $^{**}p$ <0.05; $^{***}p$ <0.01

direct effect of biased media, and it is possible one could uncouple the effect of the internet on social connectivity as opposed to enabling confirmation bias with some proxy measure for social connectivity. What is also noteworthy is that the researchers included the level of “political interest” per county as a mediating variable in parts of the analysis. So for example, if we allow f to represent the fraction of respondents in each county with such strong partisan interest, then q could represent the level of bias these agents can display due to access to partisan media on the internet. Under this interpretation we can make sense of the interaction terms in the regression results - the effect of internet access on polarisation was considerably higher for counties where political interest is higher, which is exactly what we predict from the product (fq^2) in (25).

In [16], the author argues that internet access leads to a decrease in political polarisation. This study looks solely at Twitter networks over time (but shows how they relate to political polarisation data offline). The author finds that more diverse Twitter networks lead to reduced polarisation over time. Again, our model predicts the following - since everyone is already on Twitter in this scenario, the fractions f and q are untouched. However, the author notes that more diverse networks are directly correlated with larger networks - a larger k. It follows therefore that these users with reduced polarisation experienced an increased k without a corresponding change in f or q, and the results follow.

All in all, our biased learning model has proven to provide useful insight into a long-standing debate about an important empirical topic. We show that it allows us compress a large and complex set of causal mechanisms in the literature down to the effect of three terms of interest - the prevalence of biased agents (f), degree of bias (q), and social connectivity (k). In doing so, we were able to shed insights on the mechanisms at play when it came to internet access, and provide the beginnings of a more uniform understanding of what previously conflicting data has suggested to date.

References

[1] M. H. DeGroot, Reaching a consensus. *Journal of the American Statistical Association* **69**, 118–121 (1974).

[2] H. C, Social networks and internet connectivity effects. *Information Communication and Society* **5**, 127–147 (2005).

[3] W. A. K. T. Wellman B, Smith A, Networked families, *Tech. rep.*, https://www.pewresearch.org/internet/2008/10/19/networked-families/ (2008).

[4] K. E. Lee J, Incidental exposure to news: Predictors in the social media setting and effects on information gain online. *Computers in Human behaviour* pp. 1008–1015 (2017).

[5] Y. Lelkes, G. Sood, S. Iyengar, The hostile audience: The effect of access to broadband internet on partisan affect. *American Journal of Political Science* **61**, 5–20 (2017).

[6] M. Del Vicario, A. Bessi, F. Zollo, F. Petroni, A. Scala, G. Caldarelli, H. E. Stanley, W. Quattrociocchi, The spreading of misinformation online. *Proceedings of the National Academy of Sciences* **113**, 554–559 (2016).

[7] P. Howe, A. Leiserowitz, Geographic variation in opinions on climate change at state and local scales in the usa. *Nature Climate Change* **5**, 596–603 (2015).

[8] Form 477 county data on internet access services, federal communications commission, *Tech. rep.*, https://www.fcc.gov/general/form-477-county-data-internet-access-services (2016).

[9] The geography of social capital in america, joint economic committee, *Tech. rep.*, https://www.jec.senate.gov/public/index.cfm/republicans/2018/4/the-geography-of-social-capital (2018).

[10] H. Wang, B. Wellman, Social connectivity in america: Changes in adult friendship network size from 2002 to 2007. *American Behavioral Scientist* **53**, 1148-1169 (2010).

[11] R. Putnam, P. Putnam, *Bowling Alone: The Collapse and Revival of American Community*, A Touchstone book (Simon & Schuster, 2000).

[12] D. M. K. S. Kohut A, Doherty C, A deeper partisan divide over global warming, *Tech. rep.*, https://www.people-press.org/2008/05/08/a-deeper-partisan-divide-over-global-warming/ (2008).

[13] L. Boxell, M. Gentzkow, J. M. Shapiro, Greater internet use is not associated with faster growth in political polarization among us demographic groups. *Proceedings of the National Academy of Sciences* **114**, 10612–10617 (2017).

[14] C. B, Age trends in daily social contact patterns. *Research on Aging* (2011).

[15] E. H. Berry, A. Kirschner, *Demography of Rural Aging* (2013).

[16] P. Barberá, How social media reduces mass political polarization. evidence from germany, spain, and the us. *Job Market Paper, New York University* **46** (2014).
